# *Puccinia triticina* Effector Pt3863 Targets and Subverts TaRLCK176 to Suppress Wheat Resistance to Leaf Rust

**DOI:** 10.1111/mpp.70317

**Published:** 2026-07-20

**Authors:** Jiaojie Zhao, Weishuai Bi, Na Zhang, Na Li, Xuefeng Fan, Hao Li, Lulu Song, Yue Qi, Johannes Mapuranga, Tongjun Sun, Wenxiang Yang

**Affiliations:** ^1^ College of Plant Protection Hebei Agricultural University Baoding China; ^2^ Institute of Future Agriculture Northwest Agriculture and Forestry University Yangling Shaanxi China; ^3^ Plant Protection and Quarantine Station of Hebei Province Shijiazhuang China; ^4^ Shenzhen Branch, Guangdong Laboratory for Lingnan Modern Agriculture, Genome Analysis Laboratory of the Ministry of Agriculture and Rural Affairs, Agricultural Genomics Institute at Shenzhen, Chinese Academy of Agricultural Sciences Shenzhen China; ^5^ State Key Laboratory of Plant Diversity and Specialty Crops, Institute of Botany, Chinese Academy of Sciences Beijing China

**Keywords:** effector, leaf rust, Pt3863, receptor‐like cytoplasmic kinase, wheat

## Abstract

Wheat leaf rust, caused by *Puccinia triticina*, is a widespread economically important wheat disease. During infection, *P. triticina* secretes effectors proteins to manipulate host immunity. Here we identified and characterized the *P. triticina* effector Pt3863, which is highly expressed during the early phase of infection and significantly enhances fungal virulence. We also identified the wheat receptor‐like cytoplasmic kinase TaRLCK176 as a target of Pt3863. Functional assays demonstrated that TaRLCK176 positively regulates wheat resistance against leaf rust and is required for chitin‐induced reactive oxygen species (ROS) accumulation. Pt3863 subverts this defence through a dual inhibitory mechanism: first, it suppresses TaRLCK176 phosphorylation; second, it promotes TaRLCK176 degradation via the ubiquitin‐26S proteasome pathway. Host‐induced gene silencing *Pt3863* attenuated *P. triticina* virulence, while its overexpression in transgenic wheat lines increased susceptibility to *P. tritici* . Conversely, virus‐induced gene silencing of *TaRLCK176* compromised wheat resistance. Our findings establish TaRLCK176 as a critical immune hub that positively modulates wheat resistance to leaf rust and is specifically targeted by the *P. triticina* effector Pt3863. Notably, Pt3863 has evolved a sophisticated virulence strategy to simultaneously disrupt both the phosphorylation‐mediated activation and proteasomal stability of TaRLCK176, thereby impairing host immune responses. This study elucidates a key molecular mechanism underlying the suppression of wheat immunity by *P. triticina* and highlights TaRLCK176 as a promising candidate target for the genetic engineering of durable resistance in wheat against leaf rust.

## Graphical abstract

TaRLCK176 is a critical immune hub that positively modulates wheat resistance to leaf rust and is specifically targeted by the *Puccinia triticina* effector Pt3863. Notably, Pt3863 has evolved a sophisticated virulence strategy to simultaneously disrupt both the phosphorylation‐mediated activation and proteasomal stability of TaRLCK176, thereby impairing host immune responses.

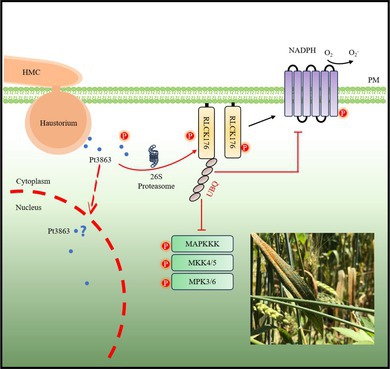

## 1 Introduction

Plants are constantly exposed to diverse biotic and abiotic stresses. Throughout the long‐term interplay between plants and pathogens, a sophisticated innate immune system has co‐evolved through their mutual adaptation. Pattern recognition receptors (PRRs) on the plant cell surface recognize pathogen‐/microbe‐associated molecular patterns (PAMPs/MAMPs) to activate pattern‐triggered immunity (PTI) (Ngou et al. 2022). PTI encompasses diverse cellular responses like calcium ions (Ca^2+^) influx, reactive oxygen species (ROS) burst, activation of mitogen‐activated protein kinase (MAPK) cascades and transcriptional reprogramming of defence genes (Godinho et al. 2025; Xu and Yang 2025). This promotes the synthesis of defence‐related proteins, plant hormones and secondary metabolites, thereby conferring broad‐spectrum resistance against pathogens (Bigeard et al. 2015). To counteract PTI, pathogens secrete effector proteins into the host cell to suppress PTI and cause disease susceptibility (Chang et al. 2024). In response, plants evolved intracellular nucleotide‐binding leucine‐rich repeat receptors (NLRs) to recognize these effectors, activating a stronger defence response termed effector‐triggered immunity (Ngou et al. 2022).

PRRs are typically receptor‐like kinases (RLKs) or receptor‐like proteins (RLPs) that lack kinase domains (Gu et al. 2017; Godinho et al. 2025), both often requiring co‐receptors to form functional receptor complexes for PAMP recognition. PAMP recognition activates these receptor complexes, which subsequently phosphorylate receptor‐like cytoplasmic kinases (RLCKs) to transduce the immune signals (Lin et al. 2013; Wang, Bai, et al. 2025). In *Arabidopsis thaliana* , the bacterial PAMP flg22 is recognized by the LRR‐RLK FLS2, which interacts with co‐receptor BAK1 to form a heterodimeric complex. This triggers a series of autophosphorylation and trans‐phosphorylation events (Chinchilla et al. 2006; Schwessinger et al. 2011; Cao et al. 2013; Sun et al. 2013), leading to the phosphorylation of RLCKs such as BIK1 and PBLs (Lin et al. 2013; Reinhardt and Leonard 2023).

BIK1 is a well‐studied member of the *Arabidopsis* RLCK‐VII subfamily. Upon PAMP perception, phosphorylated BIK1 dissociates from the receptor complex and phosphorylates the NADPH oxidase RBOHD to regulate ROS production (Li et al. 2014). The *Arabidopsis* BIK1 ortholog in rice, OsRLCK176, functions downstream of OsCERK1 in peptidoglycan and chitin signalling pathways (Ao et al. 2014). BIK1 protein stability is tightly controlled; for example, the protein kinases AtCPK28 and OsCPK4 function as negative regulators of immune responses by facilitating the breakdown of the signalling components AtBIK1 and OsRLCK176, respectively (Wang, Grubb, et al. 2018; Wang, Wang, et al. 2018). Conversely, OsCPK17 phosphorylates OsRLCK176 at Ser83, protecting it from OsPUB12‐mediated degradation, thereby enhancing rice immunity (Mou et al. 2024). Notably, multiple pathogen effectors directly target RLCK‐VII subfamily members to suppress plant immune responses (Wang, Bai, et al. 2025). For example, the *Pseudomonas syringae* effector AvrPphB cleaves RLCK BIK1/PBL1/PBL2 via its cysteine protease activity to suppress PTI (Zhang et al. 2010). The *Xanthomonas campestris* effector AvrAC uridylylates BIK1 and RIPK to reduce their kinase activity and inhibit downstream signalling (Feng et al. 2012). Furthermore, the *Phytophthora capsici* virulence factor RXLR25 targets BIK1/PBL8/PBL17 and inhibits pattern‐induced phosphorylation of RLCK‐VIIs, thereby suppressing downstream immune responses (Liang et al. 2021).

Wheat leaf rust, caused by the obligate biotrophic fungus *Puccinia triticina*, is a destructive disease responsible for severe global yield losses (Bolton et al. 2008). *P. triticina* secretes numerous effector proteins via haustoria into host cells to manipulate host defence responses (Mapuranga et al. 2025). While a few *P. triticina* effectors have been identified, such as Pt_21 which targets the thaumatin‐like protein TaTLP1 (Wang et al. 2023), Pt9029 which targets TaRCA (Chang et al. 2024) and Pt3372 which targets wheat elicitor‐responsive protein 3 (TaERP3) (Wen et al. 2025), the host components they subvert are poorly understood.

Given that RLCK‐VIIs are common targets for effectors in other pathosystems (Zhang et al. 2010; Irieda et al. 2019; Liang et al. 2021), we hypothesized that Pt3863 might suppress a member of this kinase family. Supporting this hypothesis, a recent study revealed the crucial role of TaRLCK176 in wheat resistance against stripe rust caused by *Puccinia striiformis* f. sp. *tritici* (Pst). It was demonstrated that Pst effector Pst08755 directly targets TaRLCK176 and promotes its degradation to promote infection (Wang, Du, et al. 2025). This independent finding highlights the central role of TaRLCK176 as a hub for effector‐mediated suppression across different wheat rust diseases and strengthens the rationale for exploring its function in the wheat–leaf rust pathosystem.

In this study we identified Pt3863, a highly expressed effector from *P. triticina* , which is upregulated during the early stages of infection. Pt3863 is localized to the plasma membrane and nucleus. We aimed to characterize its function in the infection process, identify its host target, and elucidate the molecular mechanisms underlying immune response suppression. Here, we report that Pt3863 interacts with and subverts TaRLCK176. We demonstrate that TaRLCK176 is essential for PTI and resistance against *P. triticina* , and Pt3863 promotes virulence by targeting TaRLCK176, inhibiting its phosphorylation and promoting its degradation via the ubiquitin‐proteasome pathway. This study reveals a mechanism by which a *P. triticina* effector, Pt3863, subverts TaRLCK176 to promote infection.

## 2 Results

### 2.1 Structural and Expression Analysis of Pt3863

We identified a highly expressed candidate effector protein, Pt3863, from a transcriptome database of virulent *P. triticina* races (KHHT, JHKT and THSN) inoculated on susceptible wheat cultivar Thatcher (Zhang et al. 2020). Sequence analysis revealed that Pt3863 encodes a secreted protein of 120 amino acids, with N‐terminal 1–19 amino acids as a signal peptide (SP). Based on effector prediction and screening criteria, Pt3863 was classified with high confidence as a candidate effector. It harbours no transmembrane domain and has no known Pfam motif. Secondary structure analysis revealed that Pt3863 contains 20.8% α‐helices, 11.7% extended chains and 67.5% random coils. Pt3863 has a theoretical isoelectric point (pI) of 8.8 and a molecular weight of 13.01 kDa, indicating that it is a small protein. The transcript level of *Pt3863* was increased during the early stages of infection, peaking at 24 h post‐inoculation (hpi), suggesting a potential role in *P. triticina* pathogenicity (Figure S1).

### 2.2 N‐Terminal SP of Pt3863 Has a Secretory Function

To verify the secretory function of the Pt3863 putative SP, we used a genetic assay based on the requirements of invertase secretion for yeast growth on sucrose or raffinose media (Jacobs et al. 1997). This assay relies on the yeast strain YTK12, which is deficient in invertase secretion and cannot grow on media containing sucrose or raffinose. The Pt3863 predicted SP (Pt3863SP) and the Avr1b (Avr1bSP) were fused to the pSUC2 vector and transformed into YTK12 (Oh et al. 2009). The positive transformants were grown on CMD−W and YRPAA plates. Similar to the positive control (Avr1bSP), the Pt3863SP‐pSUC2 enabled yeast to hydrolyse raffinose and grow on the YPRAA medium, confirming successful secretion of invertase. In contrast, transformants expressing Mg87 failed to grow on YPRAA, indicating a lack of secretion (Figure S2).

### 2.3 Pt3863 Is Localized in the Plasma Membrane and Nucleus

**FIGURE 1:**
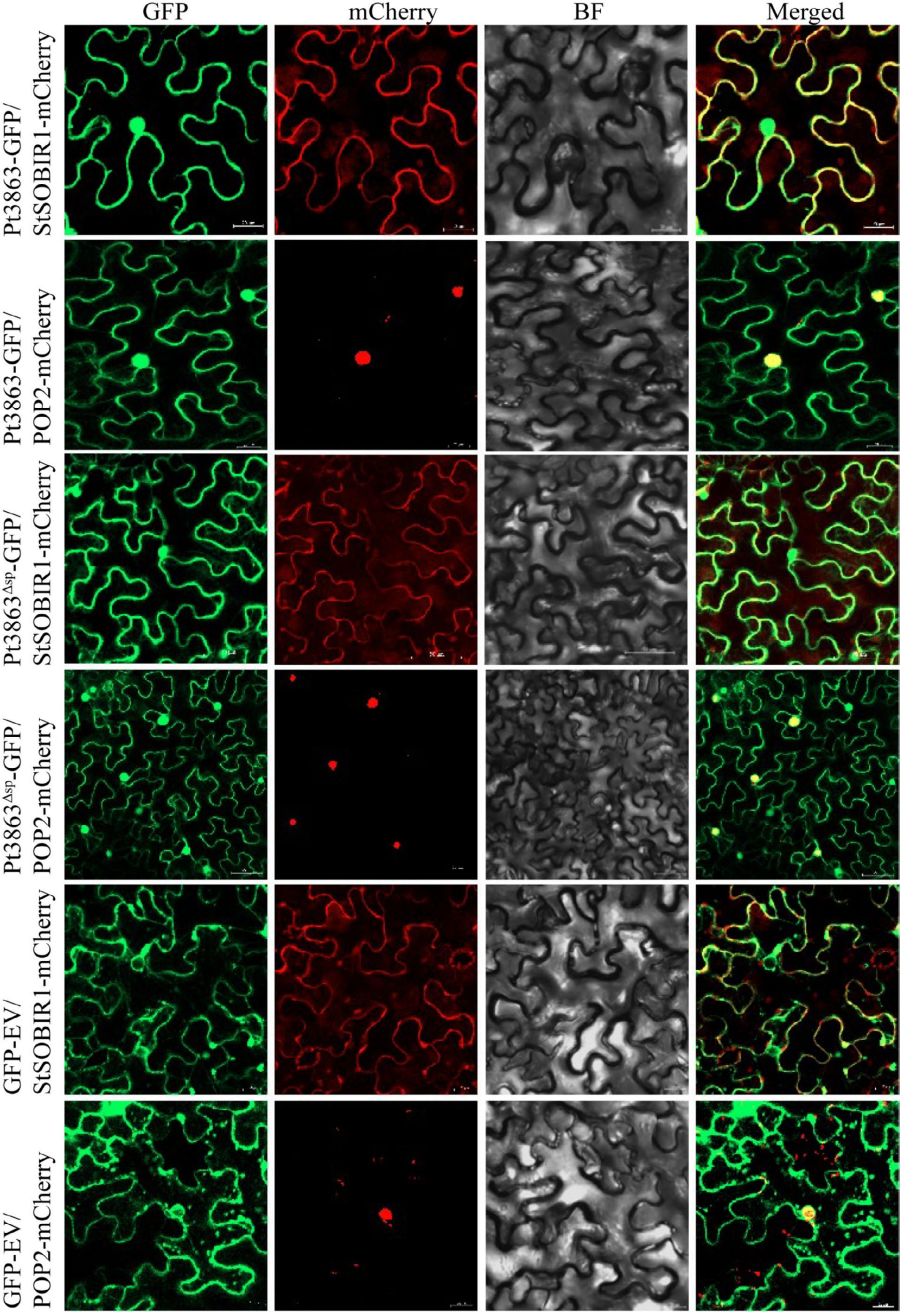
Pt3863 is localized in the plasma membrane and nucleus. Four‐week‐old fully expanded *Nicotiana benthamiana* leaves were selected, and the *Agrobacterium*‐mediated transient expression technique was employed to express Pt3863‐GFP and Pt3863^ΔSP^‐GFP fusion proteins. StSOBIR1‐mCherry was used as the plasma membrane‐localized marker, POP2‐mCherry as the nuclear‐localized marker, and GFP‐EV as the control. Scale bar represents 20 μm.

To determine its subcellular localization, we transiently co‐expressed Pt3863‐GFP or Pt3863^ΔSP^‐GFP and StSOBIR1‐mCherry or POP2‐mCherry fusion proteins in *Nicotiana benthamiana* leaves. GFP fluorescence was observed at 48 h after infiltration using a laser scanning confocal microscope. Confocal microscopy revealed that Pt3863 exhibited dual localization, targeting both the plasma membrane and the nucleus (Figure 1). To determine whether the gene is expressed normally when Pt3863 enters the cells, we used Pt3863‐GFP and Pt3863^ΔSP^‐GFP constructs and performed protein extraction for western blot analysis after transient expression in *N*. *benthamiana*. Anti‐GFP antibody detected two bands at c. 38 kDa and c. 36 kDa in *N*. *benthamiana* cells transiently expressing Pt3863‐GFP and Pt3863^ΔSP^‐GFP, respectively (Figure S3). To rule out the possibility of its cytoplasmic localization, a plasmolysis assay was performed in this study. As indicated by the red arrows in Figure S3, fluorescence signals were detected in the apoplast upon transient expression of the Pt3863‐GFP fusion protein. These results suggest that Pt3863 presence at the plasma membrane provides spatial support for a potential interaction with TaRLCK176.

### 2.4 Silencing of *Pt3863* Compromises *P. triticina* Virulence

**FIGURE 2:**
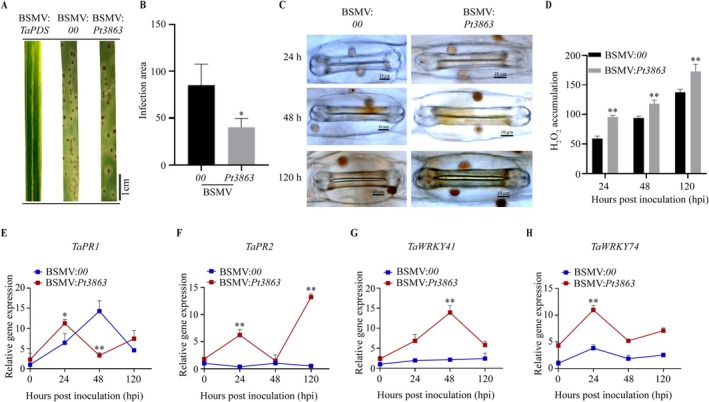
Silencing of *Pt3863* compromises *Puccinia triticina* virulence. (A) Silencing of *Pt3863* attenuates the virulence of *P. triticina* on wheat. Pathogenic phenotypes 10 days after silencing using BSMV:*Pt3863* in wheat cultivar Fielder inoculated *P. triticina* race THTT, with 12–16 biological replicates per silencing group. (B) Infection area was significantly lower than the BSMV:00 control (*p < 0.05). (C) Reactive oxygen species (ROS) accumulation after *Pt3863* silencing. ROS accumulation was observed by 3,3′‐diaminobenzidine (DAB) staining following inoculation with *P. triticina* THTT. Three time points were set for each silencing group, with 6–8 biological replicates at each time point. (D) The area measurement of H_2_O_2_ production at 24, 48 and 120 h post‐inoculation (hpi) in control and silenced group (**p < 0.01). (E–H) Relative expression levels of disease resistance‐related genes in wheat after silencing of *Pt3863*. Asterisks indicate significant differences compared with the BSMV:00 control group (*p < 0.05; **p < 0.01). Statistical analysis was conducted via Student's *t*‐test using Graphpad Prism v. 9.5, with three independent biological replicates per sample.

To explore the virulence function of Pt3863, we adopted the host‐induced gene silencing (HIGS) technology to specifically silence *Pt3863* in *P. triticina* race THTT during its infection of wheat cv. Fielder. Compared to control plants, the expression levels of *Pt3863* decreased at 24, 48 and 120 hpi in Fielder inoculated with BSMV:*Pt3863* (Figure S4), indicating the effectiveness of BSMV‐induced gene silencing. *Pt3863* silencing significantly attenuated the virulence of THTT, resulting in decreased urediniospores (Figure 2A) and decreased infection area after silencing *Pt3863* by HIGS (Figure 2B). These results demonstrate that Pt3863 plays a crucial role in fungal pathogenicity. This was accompanied by a weakened suppression of host ROS production (Figure 2C,D), and a transient upregulation of several resistance‐related genes, with significant increases observed for *TaPR1* and *TaWRKY74* at 24 hpi and for *TaWRKY41* at 48 hpi and for *TaPR2* at 120 hpi compared to the control (Figure 2E–H). These findings further demonstrate the pathogenic role of Pt3863 in the virulence of *P. triticina* . Consistent with this, we had previously found that transient expression of *Pt3863* in *N. benthamiana* suppresses flg22‐ and chitin‐induced ROS accumulation (Figure S5A,B). Protein extraction was subsequently performed for western blot analysis. Using an anti‐FLAG antibody, a specific band at c. 13 kDa was detected in *N*. *benthamiana* cells transiently expressing Pt3863‐GFP (Figure S5C). Transient expression of *Pt3863* in *N. benthamiana* also impacted flg22‐induced MAPK activation (Figure S6). Overall, these findings demonstrate that Pt3863 acts as a critical virulence factor that promotes infection by suppressing PTI responses.

### 2.5 Overexpression of *Pt3863* Attenuates the Wheat Resistance to *P. triticina*

**FIGURE 3:**
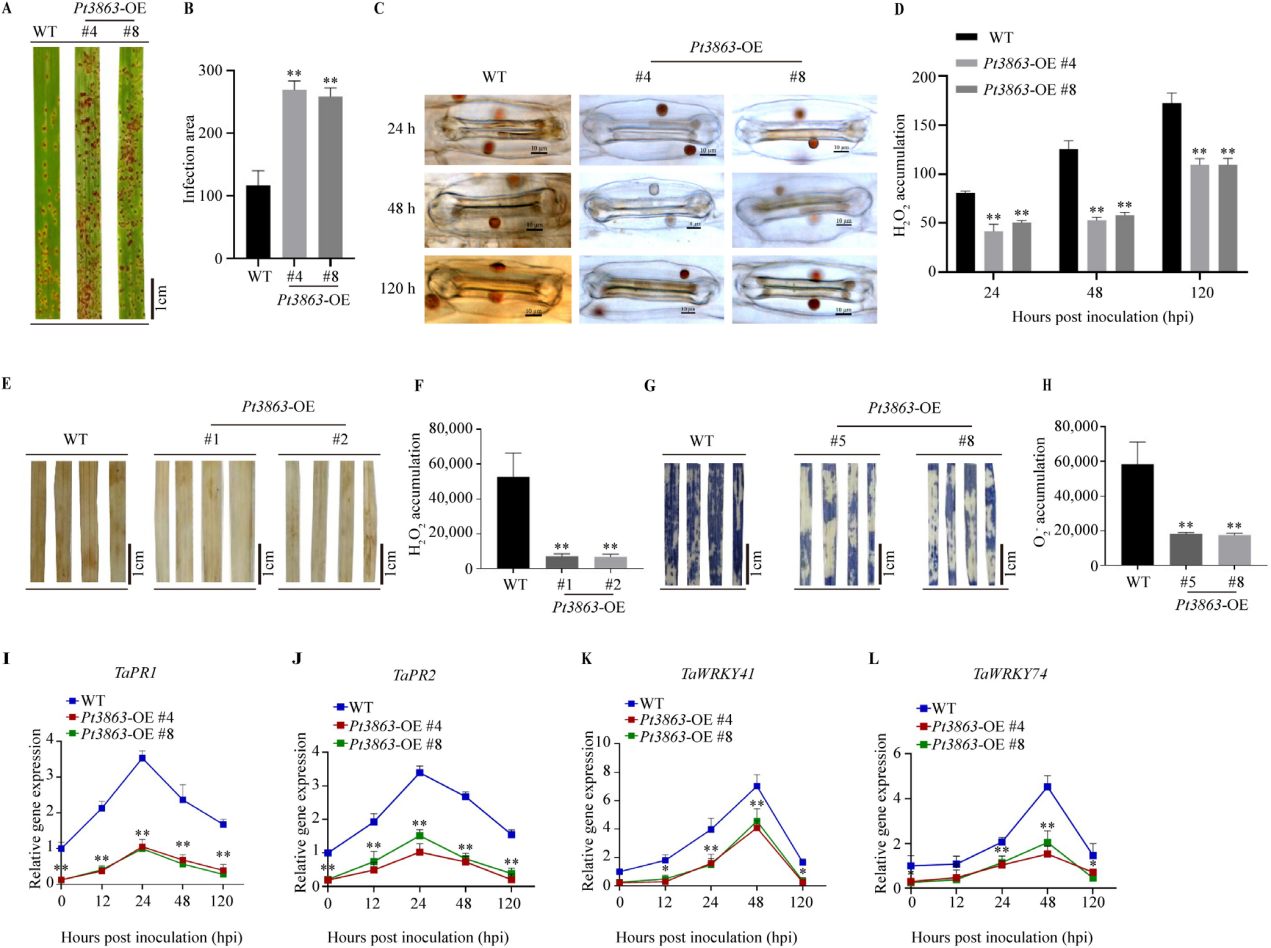
Overexpression of *Pt3863* attenuates the wheat resistance to *Puccinia triticina*. (A) The effector protein Pt3863 from *P. triticina* is involved in pathogenesis. Phenotypes of transgenic wheat *Pt3863* ^ΔSP^‐OE and wheat cultivar Fielder (WT) were evaluated inoculated with the highly virulent race THTT of *P. triticina*. Two independent experiments were performed using different transgenic lines, with 12–16 biological replicates per group. (B) Infection area was significantly higher than Fielder (**p < 0.01). (C) Reactive oxygen species (ROS) accumulation in *Pt3863* ^ΔSP^‐OE lines after inoculation with *P. triticina* THTT. ROS accumulation was observed by 3,3′‐diaminobenzidine (DAB) staining following inoculation with *P. triticina* THTT. Two independent experiments were conducted with separate lines, containing 6–8 biological replicates per line. (D) The areas of H_2_O_2_ production at 24, 48 and 120 h post‐inoculation (hpi) (**p < 0.01). (E) and (G) *Pt3863* negatively regulates chitin induced H_2_O_2_ and O_2_^−^ accumulation. (E) DAB staining was used to detect the accumulation of H_2_O_2_ in *Pt3863* ^ΔSP^‐OE transgenic wheat and wild‐type (WT) plants after chitin treatment. Two independent transgenic lines were adopted for two systematic replicates, with 12–16 biological replicates included for each line. (F) The percentage of H_2_O_2_ accumulation area relative to the total injected leaf area was analysed using ImageJ software. Asterisks indicate significant difference between transgenic lines and WT (**p < 0.01). (G) Nitroblue tetrazolium (NBT) staining was performed to detect the accumulation of O_2_^−^ in *Pt3863* ^ΔSP^‐OE transgenic wheat and WT plants after chitin treatment. Two independent transgenic lines were used for two experimental replicates, with 12–16 biological replicates per line. (H) The percentage of O_2_^−^ accumulation area relative to the total injected leaf area was analysed using ImageJ software (**p < 0.01). (I–L) Relative expression levels of disease resistance‐related genes in *Pt3863* ^*△*^^ΔSP^‐OE wheat plants after *P. triticina* inoculation. Asterisks indicate significant differences compared with the WT control group (*p < 0.05, **p < 0.01). Statistical analysis was conducted via Student's *t*‐test using Graphpad Prism v. 9.5, with three independent biological replicates per sample.

To further confirm that Pt3863 enhances virulence via inhibiting host defence responses, we obtained stable transgenic Fielder wheat lines expressing *Pt3863* ^ΔSP^‐FLAG using *Agrobacterium tumefaciens* ‐mediated transformation. Ten independent transgenic lines (OE:*Pt3863* ^ΔSP^‐FLAG) were generated, and western blot analysis confirmed that *Pt3863* ^ΔSP^‐OE was expressed at similar levels in T_1_ progeny of all 10 lines (Figure S7). Compared with wild‐type plants, *Pt3863* ^ΔSP^‐OE transgenic plants showed no obvious changes in plant height, spike length, spikelet number per spike, seed number per spike, and seed size (Figure S8A–C), indicating that Pt3863 does not affect plant growth and development. Leaves of these transgenic plants were inoculated with the *P. triticina* race THTT, and the lesion areas were recorded. Compared with the control plants, *Pt3863*‐OE exhibited a significant increase in urediniospore production, and the lesion area expanded (Figure 3A,B). The results showed that two independent *Pt3863* ^ΔSP^‐OE transgenic lines enhanced susceptibility to *P. triticina* infection.

Further analysis revealed that *Pt3863* ^ΔSP^‐OE transgenic lines exhibited reduced ROS accumulation after inoculation with *P. triticina* THTT compared to WT (Fielder wheat) (Figure 3C,D). Additionally, chitin‐triggered ROS bursts were severely inhibited in the *Pt3863* ^ΔSP^‐OE transgenic lines (Figure 3E–H), which was consistent with the transient expression results in *N. benthamiana* (Figure S5B). Next, we examined the expression of defence genes in wheat after *P. triticina* inoculation. We found that the expression of *TaPR1*, *TaPR2*, *TaWRKY41* and *TaWRKY74* was consistently lower in the *Pt3863* ^ΔSP^‐OE transgenic lines than in WT (Figure 3I–L). Collectively, overexpression of *Pt3863* suppresses wheat immune responses to *P. triticina* , thereby promoting wheat leaf rust susceptibility.

### 2.6 Pt3863 Interacts With TaRLCK176

The dual localization of Pt3863 to the plasma membrane and nucleus prompted us to investigate its potential role in disrupting PTI at the membrane level. We first established that transient expression of *Pt3863* in *N*. *benthamiana* significantly inhibited flg22‐ and chitin‐induced ROS (Figure S5A,B), indicating a broad‐spectrum inhibition of immune signalling downstream of several PRRs. To identify the Pt3863 target, we screened for physical interactions with key components of PRR complexes. Co‐immunoprecipitation (Co‐IP) assays revealed that Pt3863 interacted with the co‐receptor StSOBIR1, StCERK1 and intracellular receptor AtBIK1 (Figure S9A,B). AtBIK1 is a central positive regulator that converges signals from multiple PRRs. Based on this pronounced effect on AtBIK1 and its critical role in PTI, we prioritized it for follow‐up studies in wheat.

**FIGURE 4:**
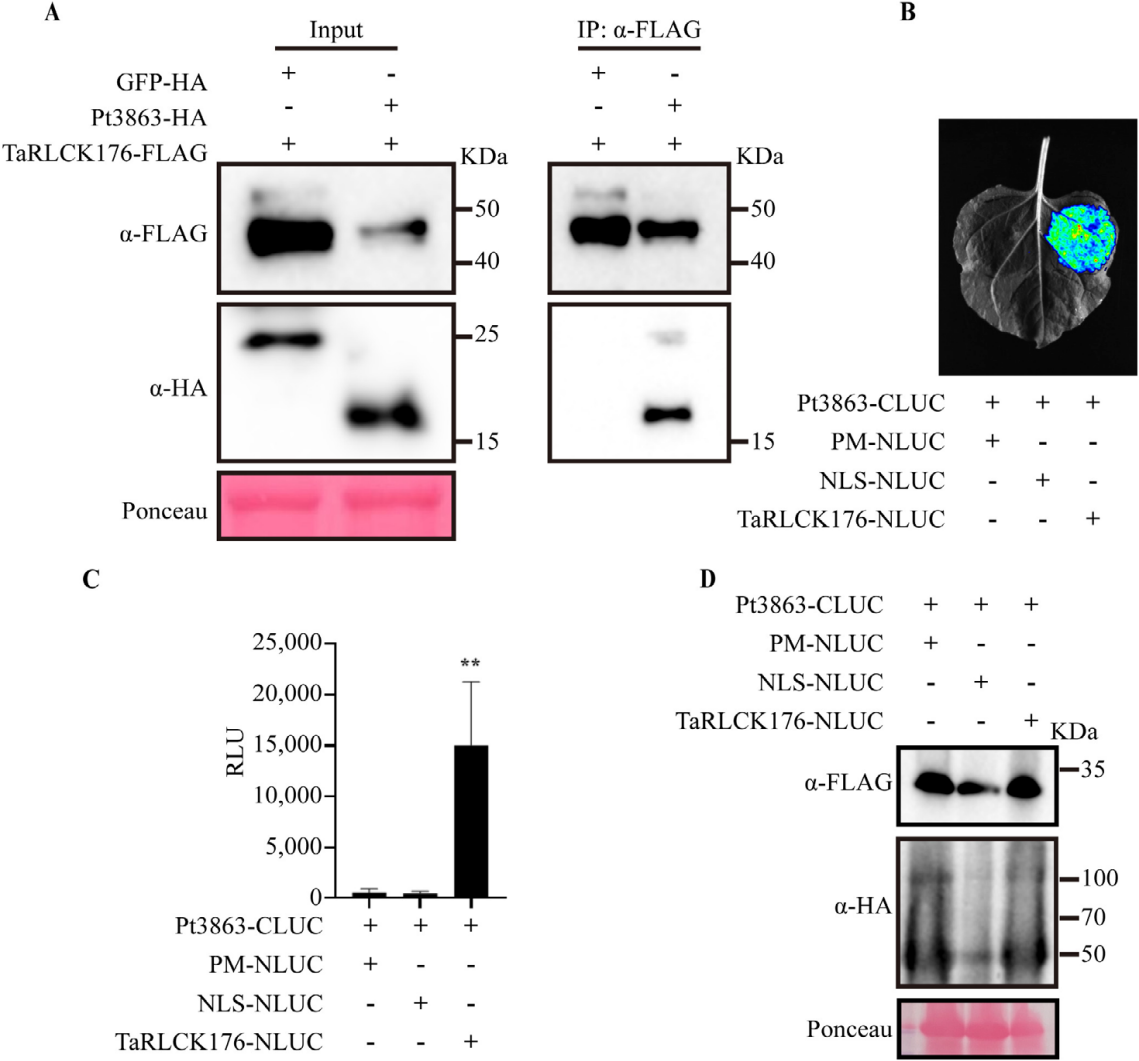
Pt3863 interacts with TaRLCK176. (A) Co‐immunoprecipitation validation of the interaction between Pt3863 and TaRLCK176. GFP‐HA/TaRLCK176‐FLAG and Pt3863‐HA/TaRLCK176‐FLAG were co‐expressed in *Nicotiana benthamiana*. Immunoprecipitation was performed using FLAG beads, and proteins were detected by western blot. (B) Split‐luciferase complementation validation of the interaction between Pt3863 and TaRLCK176. Pt3863‐Cluc and TaRLCK176‐Nluc were co‐expressed in *N. benthamiana*. LTI6b‐Nluc (a plasma membrane marker) and NLS‐Nluc (a nuclear‐localized marker) were separately used as controls. Luminescence was visualized under a fluorometer 48 h later. (C) The protein interaction intensity is shown by the relative luminescence unit (RLU) (mean ± SD, *n* ≥ 8, *n* represents sample number, **p < 0.01, Student's *t*‐test). (D) The HA and FLAG tags were fused to the Nluc and Cluc vectors, respectively. Total proteins were extracted and detected by western blot.

We identified the closest wheat homologue of AtBIK1 as TaRLCK176 through BLAST analysis, which was supported by phylogenetic analysis confirming its high sequence similarity to the well‐characterized ortholog OsRLCK176 in rice (Figure S10). The physical interaction between Pt3863 and TaRLCK176 was then confirmed in vivo using Co‐IP (Figure 4A, Figure S11) and split‐luciferase complementation assays (Figure 4B–D, Figure S12) in *N*. *benthamiana*. Intriguingly, we observed that the protein accumulation of TaRLCK176 was significantly reduced in the presence of Pt3863 (Figure 4A), suggesting that this effector may potentially compromise TaRLCK176 protein stability. These results establish TaRLCK176 as a cellular target of the *P. triticina* effector Pt3863.

### 2.7 TaRLCK176 Participates in Wheat Defence Responses Against *P. triticina*

To explore whether TaRLCK176 acts as a functional homologue of BIK1 in plant PTI, we silenced *NbBIK1* via tobacco rattle virus (TRV)‐mediated gene silencing (VIGS). Silencing of *NbBIK1* markedly inhibited flg22‐ and chitin‐triggered ROS burst (Figure S13A,B), which is consistent with the established immune function of BIK1 reported previously (Zhang et al. 2024). Further transient expression assays showed that *TaRLCK176* could restore and elevate immune‐related ROS accumulation in *NbBIK1*‐silenced leaves compared with the *GFP* control (Figure S13A,B). Because the ROS burst serves as a core early marker of PTI signalling activation, these results collectively indicate that TaRLCK176 functions downstream of PRRs and acts as a functional homologue of NbBIK1 in regulating PTI‐associated ROS production.

**FIGURE 5:**
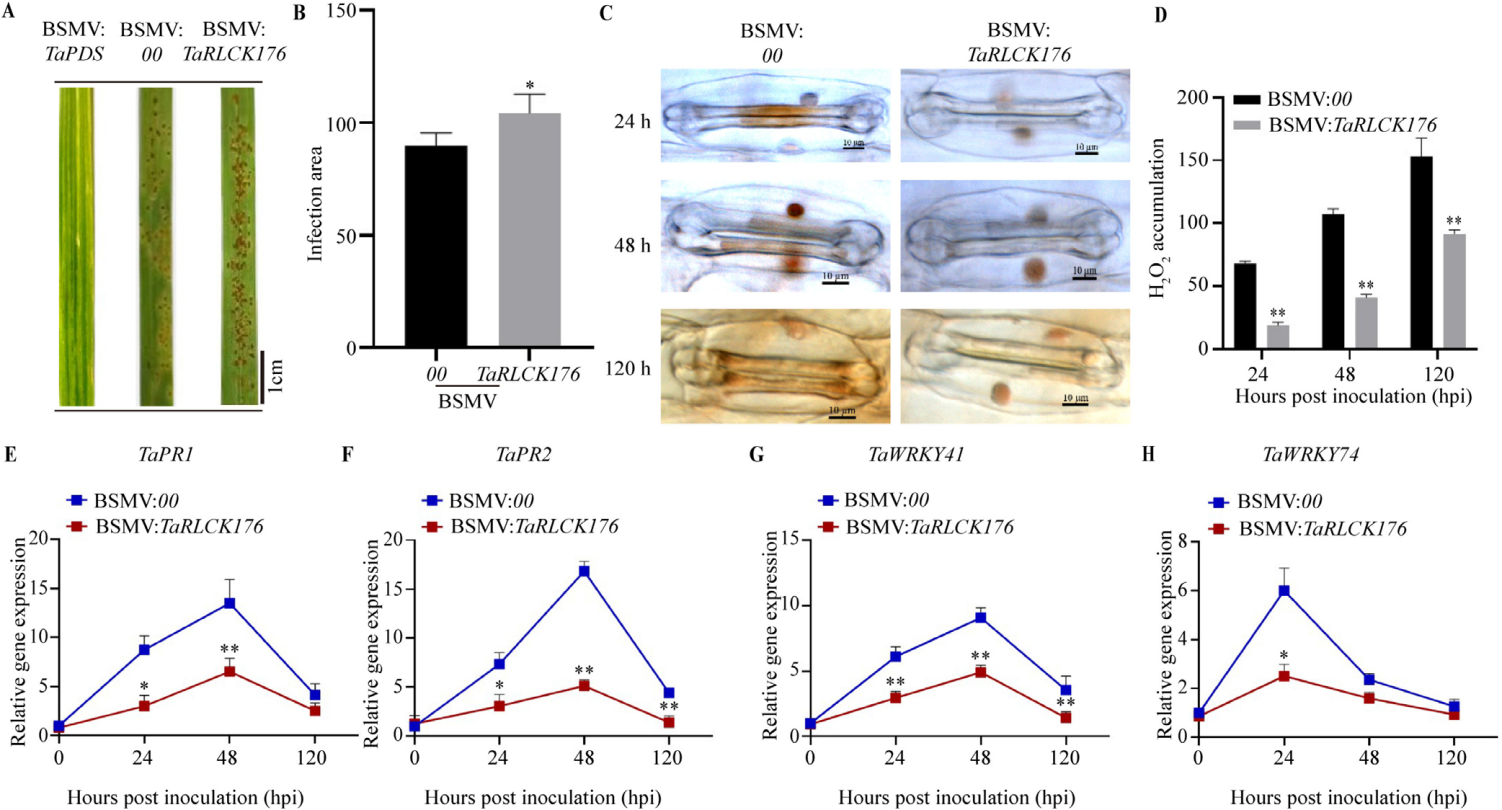
TaRLCK176 positively regulates wheat defence responses against *Puccinia triticina*. (A) Functional analysis of *TaRLCK176* silencing in wheat resistance. Wheat wild‐type (WT) plants were inoculated with *P. triticina* race THTT 10 days after barley stripe mosaic virus (BSMV) inoculation, with 12–16 biological replicates per silencing group. (B) Infection area was significantly higher than control (*p < 0.05). (C) Reactive oxygen species (ROS) accumulation after *TaRLCK176* silencing. ROS accumulation was observed and photographed using an Axio Imager M2 upright fluorescence microscope (Zeiss) after 3,3′‐diaminobenzidine (DAB) staining following inoculation with *P. triticina* THTT. Three time points were set for each silencing group, with 6–8 biological replicates at each time point. (D) The area of H_2_O_2_ production at 24, 48 and 120 h post‐inoculation (hpi) in control and silenced group (**p < 0.01). (E–H) Expression analysis of disease resistance‐related genes in *TaRLCK176‐*silenced lines after inoculation with *P. triticina* THTT (*p < 0.05, **p < 0.01). Statistical analysis was performed using Student's *t*‐test in Graphpad Prism v. 9.5 and three biological replicates were used for each sample.

To further characterize the role of TaRLCK176 in wheat resistance to leaf rust, barley stripe mosaic virus (BSMV)‐VIGS was performed to silence *TaRLCK176* in wheat cultivar Fielder. Reverse transcription‐quantitative PCR (RT‐qPCR) analysis showed a significant reduction in the transcriptional levels of *TaRLCK176* at 24, 48, and 120 h post‐inoculation (hpi) in BSMV:TaRLCK176‐silenced plants (Figure S14A,B), indicating successful silencing of *TaRLCK176*. After inoculation with *P. triticina* race THTT, we found that silencing *TaRLCK176* significantly compromised wheat resistance to leaf rust, resulting in increased urediniospores (Figure 5A,B), accompanied by reduced ROS burst during infection (Figure 5C,D). We further examined the expression of defence‐related genes during *P. triticina* infection. *TaPR1*, *TaPR2*, *TaWRKY41* and *TaWRKY74* were significantly downregulated in *TaRLCK176*‐silenced plants compared to the control (Figure 5E–H). We also analysed the expression of *TaRLCK176* in the *Pt3863* ^ΔSP^‐OE transgenic lines. The results showed that *TaRLCK176* expression was lower in the *Pt3863* ^ΔSP^‐OE transgenic lines than in WT plants from 12 to 24 hpi (Figure S15). However, by 48 hpi, expression was notably higher in the transgenic lines, which might indicate that the plant was mounting a counterdefence. These results demonstrate that TaRLCK176 participates in wheat immune responses against leaf rust.

### 2.8 Pt3863 Affects TaRLCK176 Stability and Phosphorylation

**FIGURE 6:**
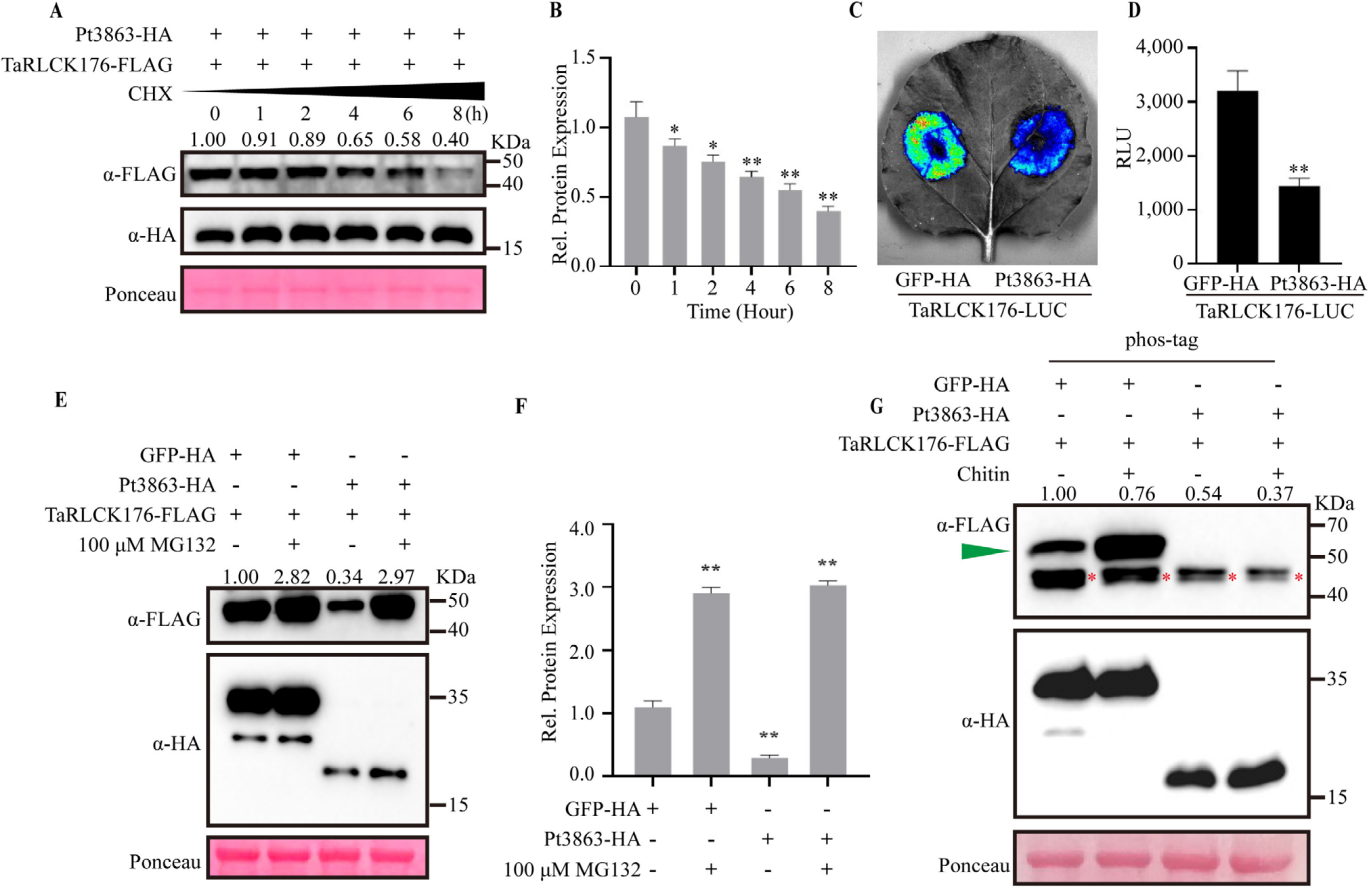
Pt3863 affects TaRLCK176 stability and phosphorylation. (A) and (B) Pt3863 reduces the protein level of TaRLCK176. Pt3863‐HA/TaRLCK176‐FLAG were co‐injected into the expanded leaves of 4‐week‐old *Nicotiana benthamiana*. After 36 h, cycloheximide (CHX) was used for treatment at the indicated time points. Proteins were extracted for western blot detection. The experiment was repeated three times with similar results, and data are mean ± SD of three independent experiments (*p < 0.05, **p < 0.01, Student's *t*‐test). (C) Constructs carrying TaRLCK176‐LUC (intact LUC) and GFP‐HA or Pt3863‐HA were co‐expressed in *N*. *benthamiana* leaves for 2 days. The infiltrated leaves were detached and sprayed with 1 mM luciferin, and the bioluminescence images were captured by a cooled CCD camera. (D) The protein interaction intensity is shown by the relative luminescence unit (RLU) (mean ± SD, *n* ≥ 8, *n* represents sample number, **p < 0.01, Student's *t*‐test). (E) and (F) Under treatment with 100 μM MG132, the protein abundance of TaRLCK176 in the presence of Pt3863 was restored. The experiments were repeated twice times with similar results and data are mean ± SD of two independent experiments (**p < 0.01, Student's *t*‐test). (G) Pt3863 inhibits chitin‐induced phosphorylation activation of TaRLCK176. TaRLCK176‐FLAG was co‐expressed with GFP‐HA or Pt3863‐HA in *N*. *benthamiana*. To eliminate the interference of ubiquitin‐mediated protein degradation, all samples were pretreated with MG132 for 6 h at 36 h post‐expression, followed by chitin treatment for 10 min. Total proteins were extracted and subjected to Phos‐tag western blot analysis. The band shift of TaRLCK176 represents its phosphorylated form, which was significantly enhanced upon chitin induction. Notably, the chitin‐triggered phosphorylation of TaRLCK176 was substantially suppressed in the presence of Pt3863, demonstrating that Pt3863 impairs the phosphorylation‐mediated activation of TaRLCK176. The experiment was repeated three times with similar results. The green arrow indicates the phosphorylated TaRLCK176 band shift, and red asterisks indicate the total protein abundance of TaRLCK176.

Given that the protein level of TaRLCK176 in the presence of Pt3863 was lower than that in the control group (Figure 4A), we hypothesized that Pt3863 may affect the protein stability of TaRLCK176. To test this, we transiently co‐expressed Pt3863‐HA/TaRLCK176 and GFP‐HA/TaRLCK176‐FLAG in *N*. *benthamiana* using *Agrobacterium*‐mediated transient gene expression. At 36 hpi, *N*. *benthamiana* plants were treated with the protein transcription and synthesis inhibitor cycloheximide (CHX) to terminate protein synthesis, and samples were harvested at 0, 1, 2, 4, 6 and 8 hpi. Total proteins were extracted, and protein accumulation was examined by western blot. The results showed that the protein accumulation level of TaRLCK176 gradually decreased compared with the control (Figure 6A,B, Figure S16). Subsequently, we fused luciferase (LUC) to the C‐terminus of TaRLCK176 (TaRLCK176‐LUC) and transiently co‐expressed it with Pt3863 in *N*. *benthamiana*. This assay showed that the luciferase signal from TaRLCK176‐LUC was significantly decreased in the presence of Pt3863 (Figure 6C,D), indicating that Pt3863 causes the degradation of TaRLCK176.

Previous studies have established that BIK1 is targeted by ubiquitin ligases including PUB25/26 and PUB4, which modulate its protein homeostasis (Fu et al. 2024). We therefore hypothesized that TaRLCK176 may also undergo degradation through the 26S proteasome pathway. Then, we transiently co‐expressed *TaRLCK176‐FLAG* with *Pt3863‐HA* or *GFP‐HA* in *N. benthamiana* and treated the plants with different concentrations of the 26S proteasome inhibitor MG132 for 6 h. We found that 1 μM MG132 treatment partially recovered the Pt3863‐triggered reduction in TaRLCK176 protein abundance (Figure S17). Upon treatment with 100 μM MG132, the protein level of TaRLCK176 in the Pt3863‐expressing group was restored to that of the GFP‐control group with MG132 treatment (Figure 6E,F), indicating that the Pt3863‐triggered degradation of TaRLCK176 is dependent on the 26S proteasome and is inhibited by MG132 treatment. Therefore, these results demonstrate that Pt3863 affects the protein stability of TaRLCK176 through the ubiquitin‐mediated protein degradation pathway.

As one of the crucial processes in protein post‐translational modification, kinase phosphorylation also modulates protein functions. It was previously demonstrated that *P. capsici* effector RXLR25 inhibits microbial pattern‐induced phosphorylation of RLCK‐VII proteins including BIK1 to suppress host immunity. Inhibition of phosphorylation blocks pattern‐induced BIK1 band shift (Liang et al. 2021). Therefore, we hypothesized that Pt3863 may affect TaRLCK176 phosphorylation. To eliminate the influence of the protein degradation pathway on phosphorylation, we used chitin as a PAMP to induce the phosphorylation of TaRLCK176 following MG132 treatment. Phosphorylation levels were then analysed in total protein extracts using phos‐tag gel. The results revealed that TaRLCK176 not only undergoes autophosphorylation but is also phosphorylated in response to chitin (Figure 6G). Intriguingly, when Pt3863 was co‐expressed with TaRLCK176, no obvious band shift was detected by western blot analysis (Figure 6G), indicating that Pt3863 inhibits both the autophosphorylation and chitin‐induced phosphorylation of TaRLCK176. Thus, we conclude that Pt3863 is likely to promote virulence by targeting TaRLCK176 and disrupt both the phosphorylation‐mediated activation and proteasomal stability of TaRLCK176.

## 3 Discussion

To counteract plant resistance, pathogens secrete an arsenal of effector proteins that manipulate critical host processes; understanding how these effectors function is crucial for elucidating pathogen virulence mechanisms and developing strategies to enhance plant resistance. In this study, we identified Pt3863 as a key pathogenicity factor, which targets and subverts RLCK TaRLCK176 to suppress wheat immunity. Multiple lines of evidence demonstrated Pt3863 as a key virulence factor. Silencing of *Pt3863* significantly attenuated *P. triticina* virulence, enhanced host ROS production, and upregulated defence‐related genes (Figure 2). Conversely, *Pt3863* overexpression in transgenic wheat enhanced susceptibility to *P. triticina* , suppressed chitin‐triggered ROS bursts, and downregulated resistance‐related genes (Figure 3). Furthermore, Pt3863's ability to suppress flg22‐ and chitin‐induced ROS and MAPK activation in *N*. *benthamiana* (Figures S5 and S6) confirms its role as a key pathogenicity factor that broadly inhibits PTI responses.

The plant immune system relies on PTI to defend against microbial pathogens, with RLCKs playing pivotal roles in transducing immune signals (Boller and Felix 2009; Dodds and Rathjen 2010; Monaghan and Zipfel 2012). In *Arabidopsis*, BIK1 is a key RLCK required for flg22‐induced RbohD phosphorylation and regulation of ROS production and stomatal immunity (Li et al. 2014), while its homologues in rice function in chitin signalling pathways (Ao et al. 2014; Li et al. 2017). Our findings demonstrate that this function is conserved in wheat (Figure 5). We observed that chitin treatment significantly promoted ROS accumulation in wheat leaves, and this response was suppressed in *TaRLCK176*‐silenced plants (Figure S18). This indicates that TaRLCK176 functions as an important component of the PTI response in wheat resistance to leaf rust.

Pathogen effectors frequently target RLCKs to manipulate plant immunity. For instance, the *P. syringae* effector HopZ1a acetylates the RLCK ZED1 (also known as HOPZ‐ETI‐DEFICIENT1 or RLCK XII‐2), triggering activation of the resistosome‐forming NLR protein ZAR1 and ETI (Bastedo et al. 2019). In this study we discovered that Pt3863 interacts with TaRLCK176, a functional wheat ortholog of BIK1, and this interaction leads to a reduction of TaRLCK176 protein accumulation (Figure 6A–D). This also aligns with the recent observation that StPM1 reduces the protein accumulation levels of StRbohC upon co‐expression (Bi et al. 2024). Accumulating evidence has demonstrated that pathogen effectors suppress host immunity by degrading resistance‐related factors (Ai et al. 2021; Bai et al. 2022; Wang, Du, et al. 2025; Ji et al. 2020; He et al. 2018). We demonstrate that Pt3863 promotes the degradation of TaRLCK176 via the ubiquitin‐mediated 26S proteosome pathway, evidenced by partial restoration of TaRLCK176 protein levels upon MG132 treatment (Figure 6E,F). This mechanism is consistent with the regulation of BIK1 by E3 ligases PUB25/26 and PUB4 in *Arabidopsis* (Fu et al. 2024), suggesting conserved immune suppression strategies across plant species. The remarkable convergence of these two studies involving different rust fungal species ( *P. triticina* and Pst) and unrelated effectors (Pt3863 and Pst08755) establishes TaRLCK176 as a common battlefield in wheat–rust interactions. This demonstrates that multiple rust fungi have independently evolved effectors to disrupt the function of the same crucial immune kinase, highlighting its importance in wheat immunity. Despite these advances, the precise E3 ligase involved in Pt3863‐mediated degradation of TaRLCK176 remains unknown, representing a crucial future research direction.

Phosphorylation is a critical process for RLCK activation (Yamaguchi et al. 2013; Ao et al. 2014; Yamada et al. 2016). Other effectors suppress PTI by inhibiting RLCK function. For example, the oomycete *P. capsici* effector RXLR25 interacts with RLCK VII members, including BIK1, PBL8 and PBL17, blocking their pattern‐induced phosphorylation and downstream PTI responses (Liang et al. 2021). Similarly, in rice, the *Xanthomonas oryzae* pv. *oryzae* effector Xoo1488 targets OsRLCK185, interfering with its phosphorylation by the chitin receptor OsCERK1 and suppressing chitin‐triggered PTI (Yamaguchi et al. 2013). We found that TaRLCK176 undergoes both autophosphorylation and chitin‐induced phosphorylation, and such phosphorylation processes are suppressed by Pt3863 (Figure 6G). Notably, much of this work was conducted in *N*. *benthamiana*. This dual strategy of destabilizing TaRLCK176 and inhibiting its activation represents a sophisticated virulence mechanism to effectively suppress PTI. Pt3863 employs a distinct mechanism compared to other rust effectors. While the unrelated effector Pst08755 also targets TaRLCK176 for 26S proteasomal degradation (Wang, Du, et al. 2025), Pt3863 adds a second layer of suppression by inhibiting TaRLCK176 phosphorylation. This functional divergence occurs despite their low (12%) amino acid sequence similarity (Figure S19), highlighting convergent evolution with distinct molecular interfaces. How Pt3863 inhibits TaRLCK176 phosphorylation awaits further investigation.

**FIGURE 7:**
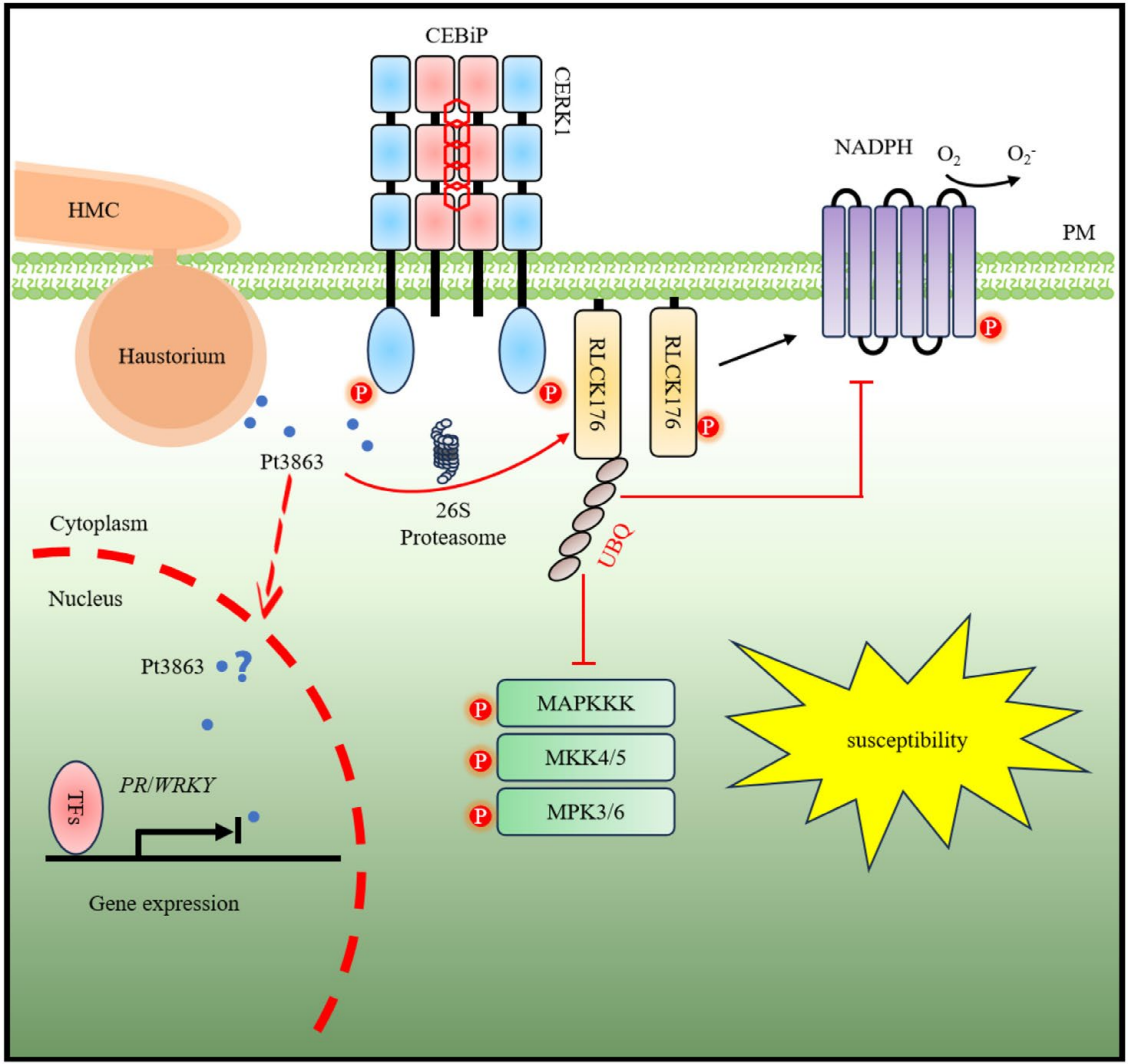
Model illustrating the mechanism by which the *Puccinia triticina* effector Pt3863 suppresses plant immunity by inhibiting TaRLCK176 activity. Upon pathogen recognition, plasma membrane‐localized PRRs, such as CERK1, phosphorylate RLCK176 to activate this central immune kinase, which orchestrates defence responses through direct regulation of NADPH oxidases to initiate reactive oxygen species (ROS) bursts and modulation of MAPK cascade activation, establishing a robust immune signalling network. *P. triticina* secretes the effector Pt3863, which exhibits dual functionality: A portion localizes to the plasma membrane to modulate TaRLCK176 phosphorylation and stability via the 26S proteasome pathway, while another portion translocate to the nucleus, though its precise molecular mechanisms remain elusive. This study focuses on the membrane‐localized effector mechanism where Pt3863 suppresses TaRLCK176 phosphorylation and mediates its degradation, inhibiting ROS accumulation and MAPK activation to facilitate *P. triticina* infection. HMC, haustorial mother cell; PM, plasma membrane; TF, transcription factor; UBQ, ubiquitin.

The dual localization of Pt3863 at the plasma membrane and the nucleus suggests potential additional modes of action. Its membrane localization aligns with the interaction with TaRLCK176, but the nuclear role is speculative and may involve targeting transcriptional regulators to broadly suppress immunity. However, this warrants further investigation. Additionally, while our results support a conserved targeting mechanism across rust species, the evolutionary and ecological implications necessitate broader phylogenetic and functional analysis. A detailed understanding of how these effectors interfere with the host ubiquitination machinery will be key to the development of strategies to protect TaRLCK176 and enhance durable rust resistance. We propose a model to explain how Pt3863 modulates TaRLCK176 activity to facilitate *P. triticina* infection in wheat (Figure 7).

In conclusion, this study elucidates a sophisticated virulence strategy employed by *P. triticina* through its effector Pt3863, which concurrently impairs phosphorylation and stability of the central immune kinase TaRLCK176. Our findings not only advance the molecular understanding of wheat– *P. triticina* interactions but also highlight TaRLCK176 as a promising target for engineered resistance.

## 4 Experimental Procedures

### 4.1 Structural and Expression Analysis of Pt3863

The sequence for the Pt3863 was screened from the transcriptome database of wheat susceptible cultivar Thatcher infected with the virulent *P. triticina* race KHHT, JHKT and THSN (https://www.ncbi.nlm.nih.gov/bioproject/PRJNA609405) (Zhang et al. 2020). Signal peptide prediction was performed by SignalP 6.0 (http://www.cbs.dtu.dk/services/SignalP/). EffectorP was used to predict whether Pt3863 was an effector or not. TMHMMv2.0 (https://services.healthtech.dtu.dk/services/TMHMM‐2.0/) was used to predict transmembrane domains within Pt3863. HMMER software was used for protein domain analysis (http://www.ebi.ac.uk/Tools/hmmer/). The Garnier‐Osguthorpe‐Robson (GOR) method (Garnier et al. 1996) was used to predict the secondary structure of Pt3863. Isoelectric point was predicted by the Expasy‐Compute pI/Mw tool (https://web.expasy.org/compute_pi/).

### 4.2 Secretory Validation

To validate the secretory function of Pt3863 signal peptide, we used the yeast signal sequence trap assay (Yin et al. 2018). The predicted signal peptide of Pt3863 was cloned into the pSUC2TZM13ORI vector (pSUC2) using specific primers (Table S2) and then transformed into the invertase‐deficient strain YTK12. YTK12 cannot grow using sucrose as its sole carbon source (Oh et al. 2009). Positive colonies were screened on CMD−W medium, and the secretion function was validated by growth on YPRAA plates containing sucrose (1% yeast extract, 2% peptone, 2% sucrose and 2 μg/mL aureobasidin A) instead of glucose (Chang et al. 2024).

### 4.3 Plant Materials and Growth Conditions

*Nicotiana benthamiana* plants were cultivated in a growth chamber at 20°C under a 16 h light/8 h dark photoperiod. Fully expanded 4‐week‐old leaves were used for assays. Wheat plants inoculated with *P. triticina* race THTT were maintained in a greenhouse at 25°C. Pathogen cultures were propagated for use in RT‐qPCR, HIGS and VIGS.

### 4.4 RT‐qPCR Analysis

Total RNA was extracted using the Sangon Biotech UNIQ‐10 Column TRIzol Total RNA Extraction Kit. For *Pt3863* expression profiling, samples were collected at 0, 12, 24, 48, 72, 96 and 120 hpi, and for HIGS and VIGS experiments samples were collected at 0, 24, 48 and 120 hpi. For overexpression experiments, samples were collected at 0, 12, 24, 48 and 120 hpi (Table S1). RNA concentration and quantification were performed using a NanoDrop One spectrophotometer (Thermo Fisher Scientific). First‐strand cDNA was synthesized using a reverse transcription kit (Beijing Lanbollide Trading Co. Ltd.). Quantitative real‐time PCR was conducted with gene‐specific primers (Table S2) and 2× RealStar Universal SYBR qPCR Mix (Beijing Konrun Chengye Biotechnology Co. Ltd.). *TaActin* was used as the reference gene, and the relative gene expression levels were calculated using the 2(^−ΔΔ*C*t^) method (Livak and Schmittgen 2001). Three biological replicates were used for each sample.

### 4.5 Plasmid Construction

Vectors for transient expression in *N. benthamiana* leaves were constructed by amplifying corresponding sequences and inserting them into the pCABIA1300 vector with different tags, including 3×FLAG, HA, GFP, NLUC and CLUC (Zhou et al. 2018). All primers used for plasmid construction are listed in Table S2. All constructs with single inserts were generated using the ClonExpress II One Step Cloning Kit (Nanjing Novozam Biotech Co. Ltd.) and verified by Sanger sequencing (Sangon Biotech (Shanghai) Co. Ltd.). For HIGS and VIGS analysis, specific cDNA fragments of *Pt3863* and *TaRLCK176* were inserted into BSMV‐γ vectors (Holzberg et al. 2002).

### 4.6 *Pt3863* Overexpression in Wheat

Transgenic wheat lines overexpressing the *Pt3863* gene (*Pt3863*‐OE) were generated in wheat cultivar Fielder. The *Pt3863* gene ORF without signal peptide was inserted into the wheat transgenic vector *pANIC6E* (*Ubi::Pt3863* ^ΔSP^) and then introduced into *A. tumefaciens* EHA105. Wheat embryos from the spring wheat variety Fielder (USDA‐ARS Germplasm Resources Information Network accession CItr 17268) were used to generate transgenic lines, with technical assistance provided by Northwest A&F University following the established protocols (Ishida et al. 2015). T_1_ seeds of *Pt3863*‐OE and wild‐type (WT) plants were germinated on moist filter paper in Petri dishes. Seedlings were transplanted into soil upon emergence of the first leaf and maintained under conditions with regular watering to sustain soil moisture. Experiments were conducted at the third‐leaf stage.

### 4.7 *Agrobacterium*‐Mediated Transient Expression

*Agrobacterium tumefaciens* GV3101 harbouring the target vector was cultured in liquid Luria Bertani (LB) medium containing rifampicin and kanamycin at 28°C with shaking. Bacterial cells were collected by centrifugation, washed twice with infiltration buffer (10 mM MgCl_2_, 10 mM MES and 200 μM acetosyringone), and resuspended in the same buffer to an optical density at 600 nm (OD_600_) of 0.5. The suspension was incubated in a 28°C incubator for 2 h and then injected into the abaxial surface of 4‐week‐old *N*. *benthamiana* leaves using a needleless disposable syringe.

### 4.8 Confocal Microscopy

Constructs fused to green fluorescent protein (GFP) were created. The *Pt3863* gene open reading frame (ORF) was cloned both with and without its signal peptide sequence into the pCABIA1300 vector, generating *35S::Pt3863‐GFP* and *35S::Pt3863* ^*ΔSP*^ *‐GFP*, respectively. All recombinant vectors were transiently expressed in *N*. *benthamiana* leaves via *Agrobacterium*‐mediated transformation, with an empty GFP vector serving as the control. Fluorescent signals were detected using a laser scanning confocal microscope LSM900 (Zeiss) 48 h after injection. GFP and RFP (mCherry, a red fluorescent protein) were imaged using 488 nm and 561 nm laser sources, respectively. The *Solanum tuberosum* suppressor of BIR1‐1 (StSOBIR1) is an LRR‐RLK primarily localized in the plasma membrane and is a reliable marker for studying protein localization (Chen et al. 2025). Pyrin‐only protein 2 (POP2) localizes to both the cytoplasm and nucleus, with notable nuclear enrichment (Periasamy et al. 2017). Therefore, StSOBIR1‐mCherry and POP2‐mCherry were used as membrane and nuclear markers, respectively.

### 4.9 Luciferase Complementation Assay

The luciferase complementation assay was performed following the protocol by Zhou et al. (2018) with slight modifications. *A. tumefaciens* GV3101 strains harbouring TaRLCK176‐NLUC and Pt3863‐CLUC constructs were mixed in a 1:1 ratio at final OD_600_ of 0.5 and then infiltrated into the abaxial side of fully expanded leaves of 4‐week‐old *N*. *benthamiana* plants. After infiltrating for 40–48 h, leaves were sprayed with 1 mM D‐luciferin and dark‐adapted for 5 min. Luminescence was then captured using a CCD imaging system (Tanon 5200). To measure luciferase activity, leaf discs were punched from infiltrated zones, placed in a 96‐well plate containing 1 mM D‐luciferin, and analysed for luminescence using a Multi‐Mode microplate reader (SpectraMax iD3).

### 4.10 Co‐IP Assays

*Agrobacterium tumefaciens* GV3101 strains carrying *Pt3863‐HA* (pCABIA1300) and *TaRLCK176‐FLAG* (pCABIA1300) were mixed in a 1:1 ratio and infiltrated into *N*. *benthamiana* leaves. 48 h after infiltration, the leaves were collected for total protein extraction. Protein purification was performed using DYKDDDDK‐Nanoab‐Magnetic (Beijing Lanbollide, PFM025) at 4°C with rotation for 2 h. The beads were then separated using a magnetic rack and washed by resuspending them in 500 μL of the wash buffer, followed by magnetic separation at 4°C and washing twice. Beads were then treated with 2× SDS‐PAGE loading buffer at 100°C for 10 min to elute proteins. For immunodetection, the corresponding anti‐HA antibody (1: 1000, E‐AB‐40523; Elabscience Biotechnology Co. Ltd.) and anti‐FLAG antibody (1: 1000, E‐AB‐40525; Elabscience Biotechnology Co. Ltd.) were used along with a goat anti‐rabbit IgG‐horseradish peroxidase conjugate antibody (1: 5000, S0101; LABLEAD). Immunoblotting was carried out to detect the precipitated proteins and crude protein (input).

### 4.11 ROS Assays

The third leaves of transgenic wheat (*Pt3863* ^ΔSP^‐OE) and WT plants were infiltrated with 10 μg/mL chitin. At 2 h post‐injection, leaves were harvested and immersed in staining solutions containing 3,3′‐diaminobenzidine (DAB) or nitroblue tetrazolium (NBT) for 24 h, then decolourized in boiling acetic acid:ethanol (1:1, v/v) until chlorophyll was cleared. Brown (DAB) or blue (NBT) deposits indicate ROS accumulation.

*Agrobacterium tumefaciens* GV3101 carrying Pt3863‐GFP (pCABIA1300) was expressed in *N*. *benthamiana* leaves. The leaf discs were collected from *N*. *benthamiana* leaves at 48 h post‐agroinfiltration and incubated with 100 μL double‐distilled water in a 96‐well plate for 12 h to eliminate the wounding effect. Water was replaced by 100 μL of a reaction solution (20 μM luminol, 10 μg/mL horseradish peroxidase), supplemented with 10 μg/mL chitin (Sangon Biotech) or 1 μM flg22 (Sangon Biotech). Luminescence was measured with a Tecan F200 microplate luminometer for a period of 30 min. ROS production was indicated as the mean of relative light units (RLU).

### 4.12 TRV VIGS in *N. benthamiana*

About 300 bp sequence of target genes was selected to generate the pTRV‐RNA2 plasmid. The pTRV‐RNA1 and pTRV‐RNA2 plasmids were transformed into *A. tumefaciens* GV3101. Bacterial cultures were prepared as previously described by Velásquez et al. (2009). Briefly, cells were pelleted by 3000 *g* centrifugation, resuspended in a solution containing 10 mM MgCl_2_, 10 mM 2‐(*N*‐morpholino) ethanesulfonic acid (MES) and 200 μM acetosyringone, adjusted to an optical density at 600 nm (OD_600_) of 0.3 and incubated at 25°C for at least 3 h. Bacterial cultures containing pTRV‐RNA1 and pTRV‐RNA2 derivatives were mixed at a 1:1 ratio and inoculated into the true leaves of 2‐week‐old plants using a needleless syringe. After infiltration, the plants were kept at 20°C–22°C in a growth chamber with a 16 h day length and 50% relative humidity for at least three and a half weeks before using them for assays.

### 4.13 BSMV VIGS in Wheat

Gene‐specific silencing fragments for *Pt3863* and *TaRLCK176* were designed from cDNA sequences using siFi21 software with default parameters to minimize off‐target effects. Silencing fragments were cloned and inserted into BSMV. The BSMV components α and β were mixed in a ratio of 1:1:1 with either BSMV:γ or a recombinant γ‐gene and then propagated on 2nd leaves of 4‐week‐old *N*. *benthamiana* via *Agrobacterium* injection (Yuan et al. 2011). Systemically infected *N*. *benthamiana* leaves were collected 12 days post‐agroinfiltration, homogenized in 0.1 M phosphate buffer, and mechanically inoculated onto two‐leaf‐stage wheat by gentle abrasion with carborundum. BSMV:00 (empty γ vector) and BSMV:*PDS* (phytoene desaturase) served as negative and positive silencing controls, respectively. At 10 days post‐BSMV inoculation, wheat plants were inoculated with *P. triticina* race THTT. Disease phenotypes (uredinia density/area) were assessed 14 days post‐fungal inoculation. Silencing efficiency was confirmed via RT‐qPCR.

### 4.14 Analysis of the Effect of Effector Protein on the Phosphorylation Activity of Target Protein

Pt3863‐HA and TaRLCK176‐FLAG were transiently co‐expressed in *N*. *benthamiana*, with GFP‐HA and TaRLCK176‐FLAG serving as the control group. At 36 h post‐expression, the leaves were treated with 1 μM MG132. After 6 h of treatment, 10 μg/mL chitin was applied for 10 min, and total proteins were extracted. For the preparation of Phos‐tag SDS‐PAGE gels, apart from standard SDS‐PAGE components, 10 mM MnCl_2_ (100 μL per 10 mL gel solution) and 20 μL of 5 mM phos‐tag acrylamide solution were supplemented additionally. After electrophoresis, the gels were washed three times for 20 min each with 1× transfer buffer containing 10 mM EDTA to remove metal ion chelation. All subsequent operations were carried out following standard western blot protocols.

## Author Contributions

**Jiaojie Zhao:** investigation, writing – original draft, methodology, validation, conceptualization, formal analysis, visualization, resources. **Weishuai Bi:** investigation, methodology, validation, conceptualization. **Na Zhang:** writing – review and editing. **Yue Qi:** validation. **Johannes Mapuranga:** writing – review and editing. **Lulu Song:** validation. **Wenxiang Yang:** conceptualization, funding acquisition, supervision. **Hao Li:** validation. **Na Li:** funding acquisition. **Tongjun Sun:** writing – review and editing. **Xuefeng Fan:** writing – review and editing.

## Funding

The authors declare that financial support was received for the research and publication of this article. This research was financially supported by the Natural Science Foundation of China (32172367 and 31571956), Natural Science Foundation of Hebei Province (C2020204071 and C2024204135), Hebei Province Innovation Team of Modern Agricultural Industry Technology System (HBCT2023010204), and the AGIS Talents Start‐up Research Fund to TS.

## Conflicts of Interest

The authors declare no conflicts of interest.

## Supporting information


**Figure S1:** The expression profile of *Pt3863*.

**Figure S2:** Functional validation of the N‐terminal signal peptide of Pt3863 using the yeast invertase secretion assay.

**Figure S3:** Plasmolysis verifies the subcellular localization of Pt3863 .

**Figure S4:** The silencing efficiency of *Pt3863* was assessed using reverse transcription‐quantitative PCR in *Pt3863*‐silenced plants.

**Figure S5:** Pt3863 suppresses flg22‐ and chitin‐induced reactive oxygen species (ROS).

**Figure S6:** Pt3863 can suppress flg22 induced MAPK activation.

**Figure S7:** Protein detection of Pt3863^ΔSP^ transgenic wheat.

**Figure S8:** Agronomic traits of *Pt3863*‐OE transgenic wheat lines.

**Figure S9:** Pt3863 interacts with key components of PRR complexes.

**Figure S10:** Phylogenetic analysis of RLCK homologues from wheat and other species.

**Figure S11:** Co‐immunoprecipitation validation of the interaction between Pt3863 and TaRLCK176.

**Figure S12:** Split‐luciferase complementation validation of the interaction between Pt3863 and TaRLCK176.

**Figure S13:** Silencing of *NbBIK1* inhibited flg22‐ and chitin‐induced reactive oxygen species (ROS), while transient expression of *TaRLCK176* restored ROS accumulation.

**Figure S14:** The silencing fragment and silencing efficiency of *TaRLCK176*.

**Figure S15:** Expression analysis of *TaRLCK176* genes in *Pt3863* ^*△SP*^‐OE transgenic wheat during *Puccinia triticina* infection.

**Figure S16:** TaRLCK176 exhibits no natural degradation.

**Figure S17:** Pt3863 affects the protein stability of TaRLCK176 through the ubiquitin mediated protein degradation pathway.

**Figure S18:** TaRLCK176 positively regulates chitin‐induced reactive oxygen species (ROS) accumulation.

**Figure S19:** Sequence alignment of Pt3863 and Pst08755.

**Table S1:** RNA extraction time points.

**Table S2:** The primers used in the study.

## Data Availability

The data that support the findings of this study are available from the corresponding author upon reasonable request.
