# Supplementary material for: Puccinia triticina Effector Pt3863 Targets and Subverts TaRLCK176 to Suppress Wheat Resistance to Leaf Rust
Source: Mol Plant Pathol. 2026 Jul 20;27(7):e70317. doi: 10.1111/mpp.70317 (PMC13382533; doi:10.1111/mpp.70317)
Supplement: Supplementary file 1 — Figure S1: The expression profile of Pt3863. [file MPP-27-e70317-s009.docx]

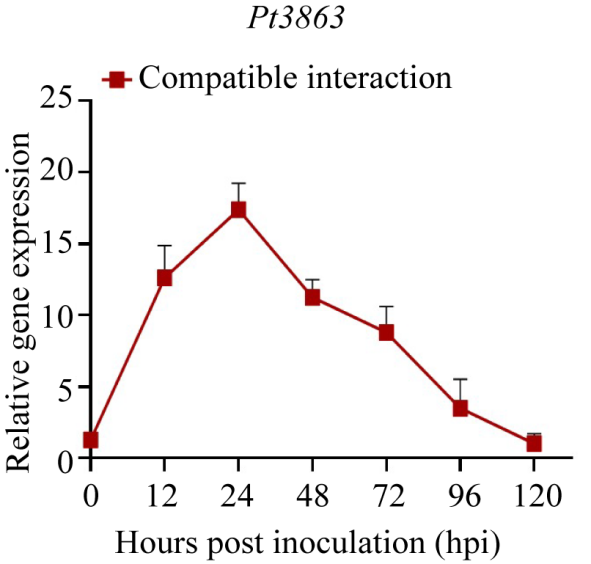


**Supplementary Figure 1. The expression profile of *Pt3863*.**

Wheat leaves inoculated with *Puccinia* *triticina* (*Pt*) were used to extract RNA, and qPCR was performed to detect *Pt3863* transcription levels using *TaActin* as the reference gene (mean ± se). Three biological replicates were used for each sample.
