# Supplementary material for: Puccinia triticina Effector Pt3863 Targets and Subverts TaRLCK176 to Suppress Wheat Resistance to Leaf Rust
Source: Mol Plant Pathol. 2026 Jul 20;27(7):e70317. doi: 10.1111/mpp.70317 (PMC13382533; doi:10.1111/mpp.70317)
Supplement: Supplementary file 2 — Figure S2: Functional validation of the N‐terminal signal peptide of Pt3863 using the yeast invertase secretion assay. [file MPP-27-e70317-s003.docx]

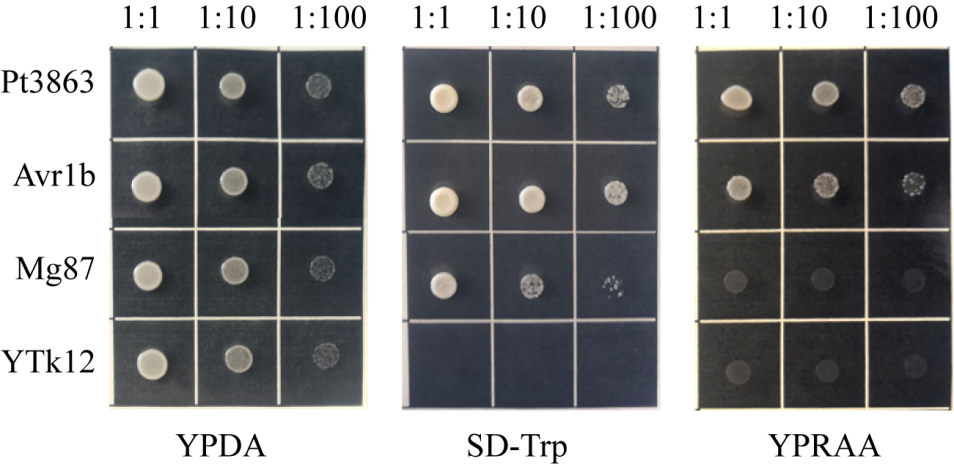


**Supplementary Figure 2.** **Functional validation of the N-terminal signal peptide of Pt3863 using the yeast invertase secretion assay.**

The signal peptides of Mg87, Avr1b and Pt3863 were fused into pSuc2t7M13ori vector, and the resulting recombinant plasmid were transformed into yeast strain YTK12. YTK12 carrying pSUC2-Avr1b served as positive control, and YTK12 and YTK12 carrying pSUC2-Mg87 were used as negative control. The yeast transformants were cultured on CMD-W medium, which promotes yeast growth without secretion of the transformed enzyme, as well as on YPRAA medium, facilitating yeast growth with secretion of the invertase enzyme.
