# Supplementary material for: Puccinia triticina Effector Pt3863 Targets and Subverts TaRLCK176 to Suppress Wheat Resistance to Leaf Rust
Source: Mol Plant Pathol. 2026 Jul 20;27(7):e70317. doi: 10.1111/mpp.70317 (PMC13382533; doi:10.1111/mpp.70317)
Supplement: Supplementary file 3 — Figure S3: Plasmolysis verifies the subcellular localization of Pt3863 . [file MPP-27-e70317-s001.docx]

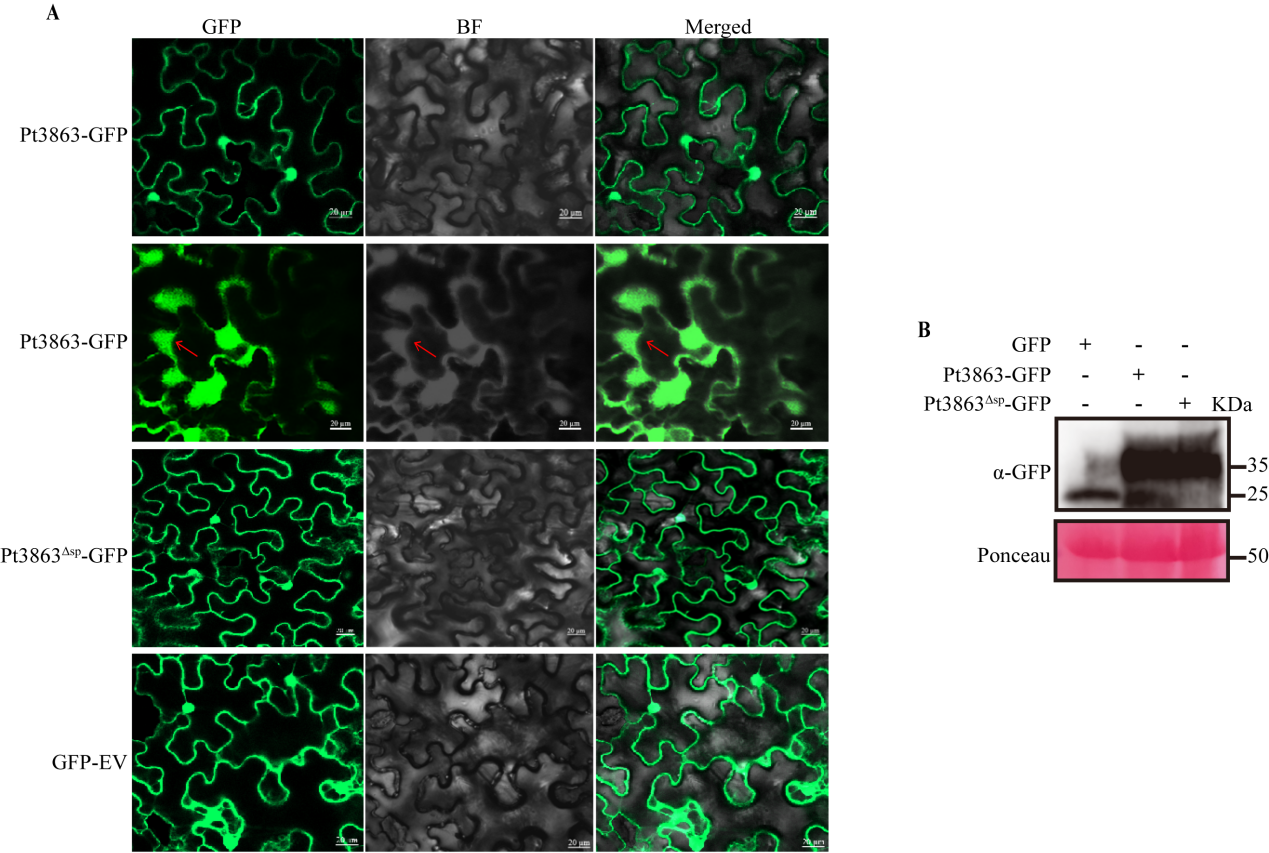


**Supplementary Figure 3. Plasmolysis verifies the subcellular localization of Pt3863.**

A: Pt3863 protein was localized in the apoplastic space. The *Agrobacterium*-mediated transient expression system was employed to express the Pt3863-GFP, Pt3863^ΔSP^-GFP fusion proteins and GFP-EV in N. benthamiana. The plasmolysis experiment of tobacco leaves expressing Pt3863‑GFP was harvested and incubated in 800 mM sorbitol solution for 6 min to induce plasmolysis. The plasmolysis was observed and photographed, with the plasma membrane indicated by red arrows; B: Western blot detection of protein expression. Anti-GFP antibody (1: 5000, HT801; TransGen Biotech, Beijing, China) were used along with a goat anti-mouse IgG-horseradish peroxidase conjugate antibody (1: 10000, HS201; TransGen Biotech).
