# Supplementary material for: Puccinia triticina Effector Pt3863 Targets and Subverts TaRLCK176 to Suppress Wheat Resistance to Leaf Rust
Source: Mol Plant Pathol. 2026 Jul 20;27(7):e70317. doi: 10.1111/mpp.70317 (PMC13382533; doi:10.1111/mpp.70317)
Supplement: Supplementary file 4 — Figure S4: The silencing efficiency of Pt3863 was assessed using reverse transcription‐quantitative PCR in Pt3863‐silenced plants. [file MPP-27-e70317-s013.docx]

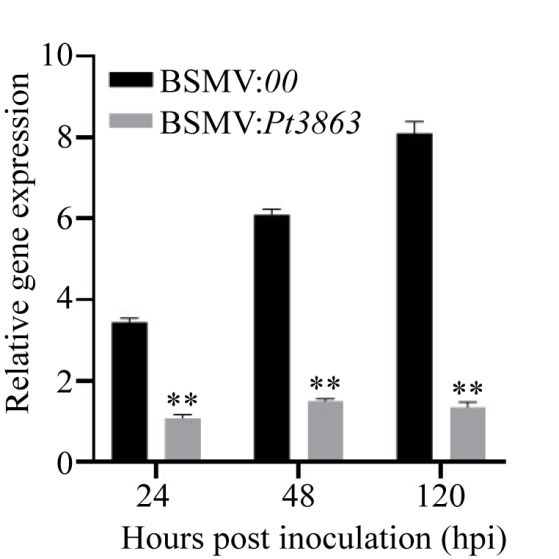


**Supplementary Figure 4. The silencing efficiency of *Pt3863* was assessed using qRT-PCR in *Pt3863*-silenced plants.**

Statistical analysis was performed using Student’s *t*-test in Prism v9.5 and three biological replicates were used for each sample ( ** *p* < 0.01).
