# Supplementary material for: Puccinia triticina Effector Pt3863 Targets and Subverts TaRLCK176 to Suppress Wheat Resistance to Leaf Rust
Source: Mol Plant Pathol. 2026 Jul 20;27(7):e70317. doi: 10.1111/mpp.70317 (PMC13382533; doi:10.1111/mpp.70317)
Supplement: Supplementary file 5 — Figure S5: Pt3863 suppresses flg22‐ and chitin‐induced reactive oxygen species (ROS). [file MPP-27-e70317-s011.docx]

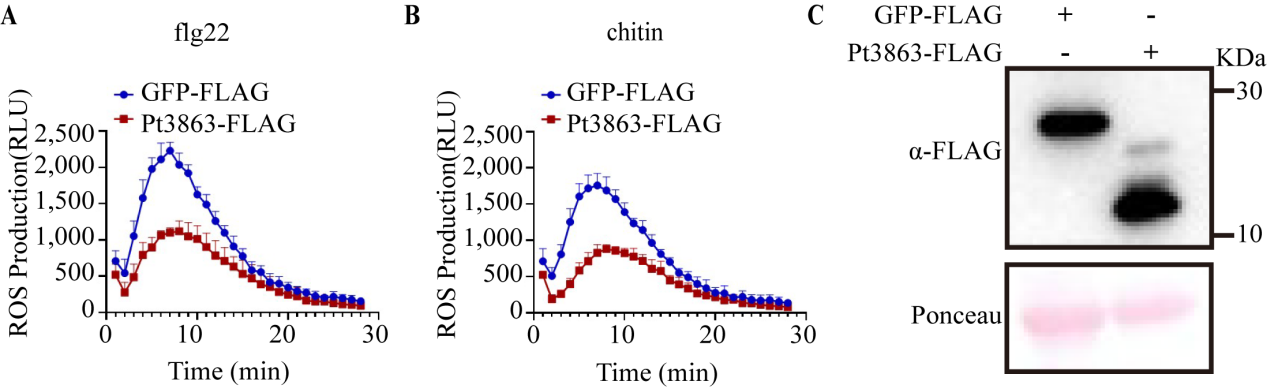


**Supplementary Figure 5. Pt3863 suppresses flg22- and chitin-induced ROS.**

A: Pt3863 suppresses flg22- induced ROS; B: Pt3863 suppresses chitin-induced ROS. Pt3863-FLAG fusion protein was transiently expressed in *N*. *benthamiana*, treated with buffers containing chitin and flg22, and read by a microplate reader; C: After transient expression of GFP-FLAG and Pt3863-FLAG in *N*. *benthamiana*, followed by protein extraction and western blot detection with anti-FLAG antibody.
