# Supplementary material for: Puccinia triticina Effector Pt3863 Targets and Subverts TaRLCK176 to Suppress Wheat Resistance to Leaf Rust
Source: Mol Plant Pathol. 2026 Jul 20;27(7):e70317. doi: 10.1111/mpp.70317 (PMC13382533; doi:10.1111/mpp.70317)
Supplement: Supplementary file 6 — Figure S6: Pt3863 can suppress flg22 induced MAPK activation. [file MPP-27-e70317-s004.docx]

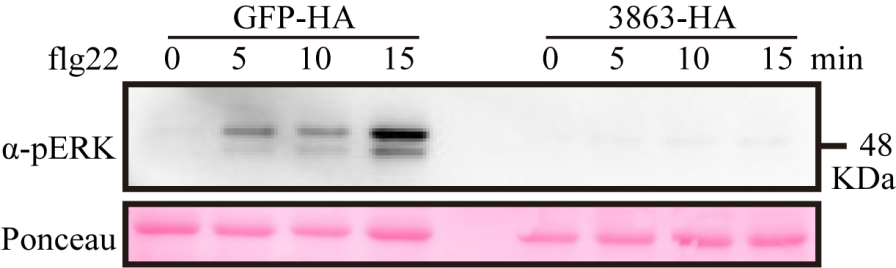


**Supplementary Figure 6. Pt3863 can suppress flg22 induced MAPK activation.**

After transient expression of GFP-HA and Pt3863-HA in *N*. *benthamiana*, treatment with 1 μM flg22 was performed for 0, 5, 10, 15 min, followed by protein extraction and MAPK signal detection by western blot with anti-pERK antibody.
