# Supplementary material for: Puccinia triticina Effector Pt3863 Targets and Subverts TaRLCK176 to Suppress Wheat Resistance to Leaf Rust
Source: Mol Plant Pathol. 2026 Jul 20;27(7):e70317. doi: 10.1111/mpp.70317 (PMC13382533; doi:10.1111/mpp.70317)
Supplement: Supplementary file 7 — Figure S7: Protein detection of Pt3863ΔSP transgenic wheat. [file MPP-27-e70317-s005.docx]

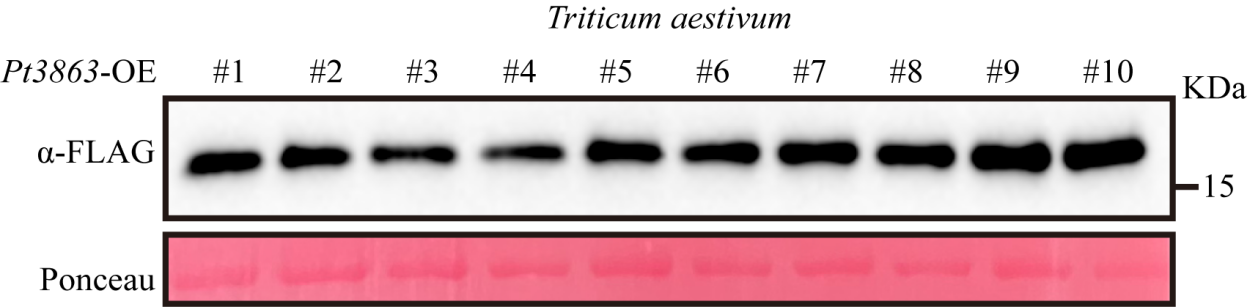


**Supplementary Figure 7. Protein detection of *Pt3863*^ΔSP^ transgenic wheat.**

Total proteins were extracted from wheat lines *Pt3863*^ΔSP^-OE #L1 to #L10, and immunoblotted with anti-FLAG antibody for Pt3863^△sp^-FLAG protein.
