# Supplementary material for: Puccinia triticina Effector Pt3863 Targets and Subverts TaRLCK176 to Suppress Wheat Resistance to Leaf Rust
Source: Mol Plant Pathol. 2026 Jul 20;27(7):e70317. doi: 10.1111/mpp.70317 (PMC13382533; doi:10.1111/mpp.70317)
Supplement: Supplementary file 8 — Figure S8: Agronomic traits of Pt3863‐OE transgenic wheat lines. [file MPP-27-e70317-s017.docx]

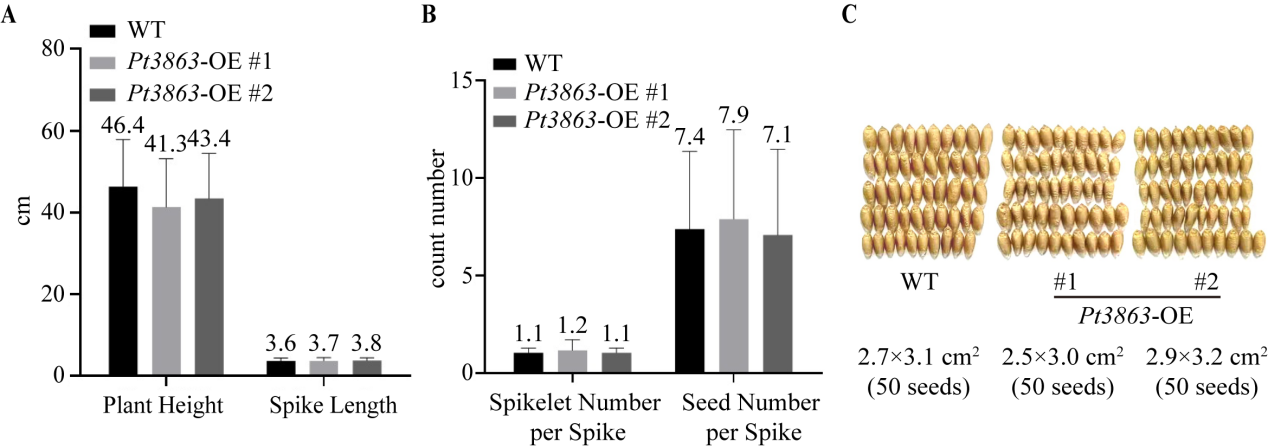


**Supplementary Figure 8. Agronomic traits of *Pt3863*-OE transgenic wheat lines.**

(A) Plant height, spike length, (B) number of spikelets, number of grains per spike, and (C) seed size of wheat transgenic materials *Pt3863*-OE and wild-type (WT) were investigated under greenhouse conditions. Independent families were used, each including 15-20 biological replicates.
