# Supplementary material for: Puccinia triticina Effector Pt3863 Targets and Subverts TaRLCK176 to Suppress Wheat Resistance to Leaf Rust
Source: Mol Plant Pathol. 2026 Jul 20;27(7):e70317. doi: 10.1111/mpp.70317 (PMC13382533; doi:10.1111/mpp.70317)
Supplement: Supplementary file 9 — Figure S9: Pt3863 interacts with key components of PRR complexes. [file MPP-27-e70317-s019.docx]

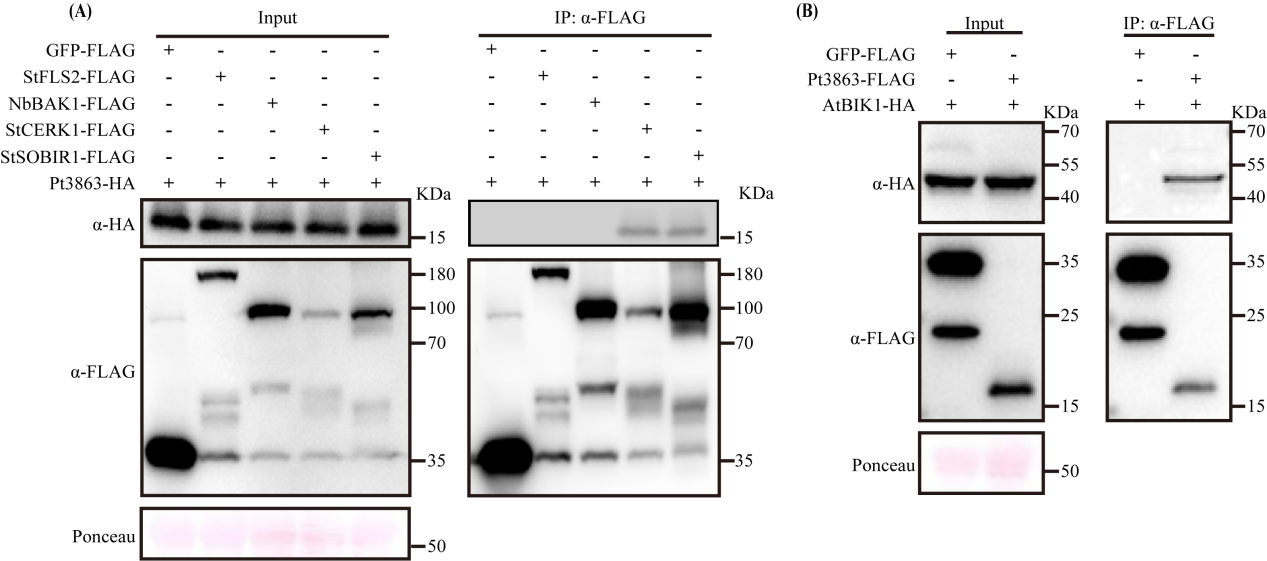


**Supplementary Figure 9. Pt3863 interacts with key components of PRR complexes.**

Co-IP assays revealed that Pt3863 interacts with the (A) StSOBIR1, StCERK1, and (B) AtBIK1. GFP-FLAG/Pt3863-HA, StFLS2-FLAG/Pt3863-HA, NbBAK1-FLAG/Pt3863-HA, StCERK1-FLAG/Pt3863-HA, StSOBIR1-FLAG/Pt3863-HA, and Pt3863-FLAG/AtBIK1-HA were co-expressed in *N*. *benthamiana*. Co-IP was performed using FLAG beads, followed by protein detection via western blot
