# Supplementary material for: Puccinia triticina Effector Pt3863 Targets and Subverts TaRLCK176 to Suppress Wheat Resistance to Leaf Rust
Source: Mol Plant Pathol. 2026 Jul 20;27(7):e70317. doi: 10.1111/mpp.70317 (PMC13382533; doi:10.1111/mpp.70317)
Supplement: Supplementary file 10 — Figure S10: Phylogenetic analysis of RLCK homologues from wheat and other species. [file MPP-27-e70317-s002.docx]

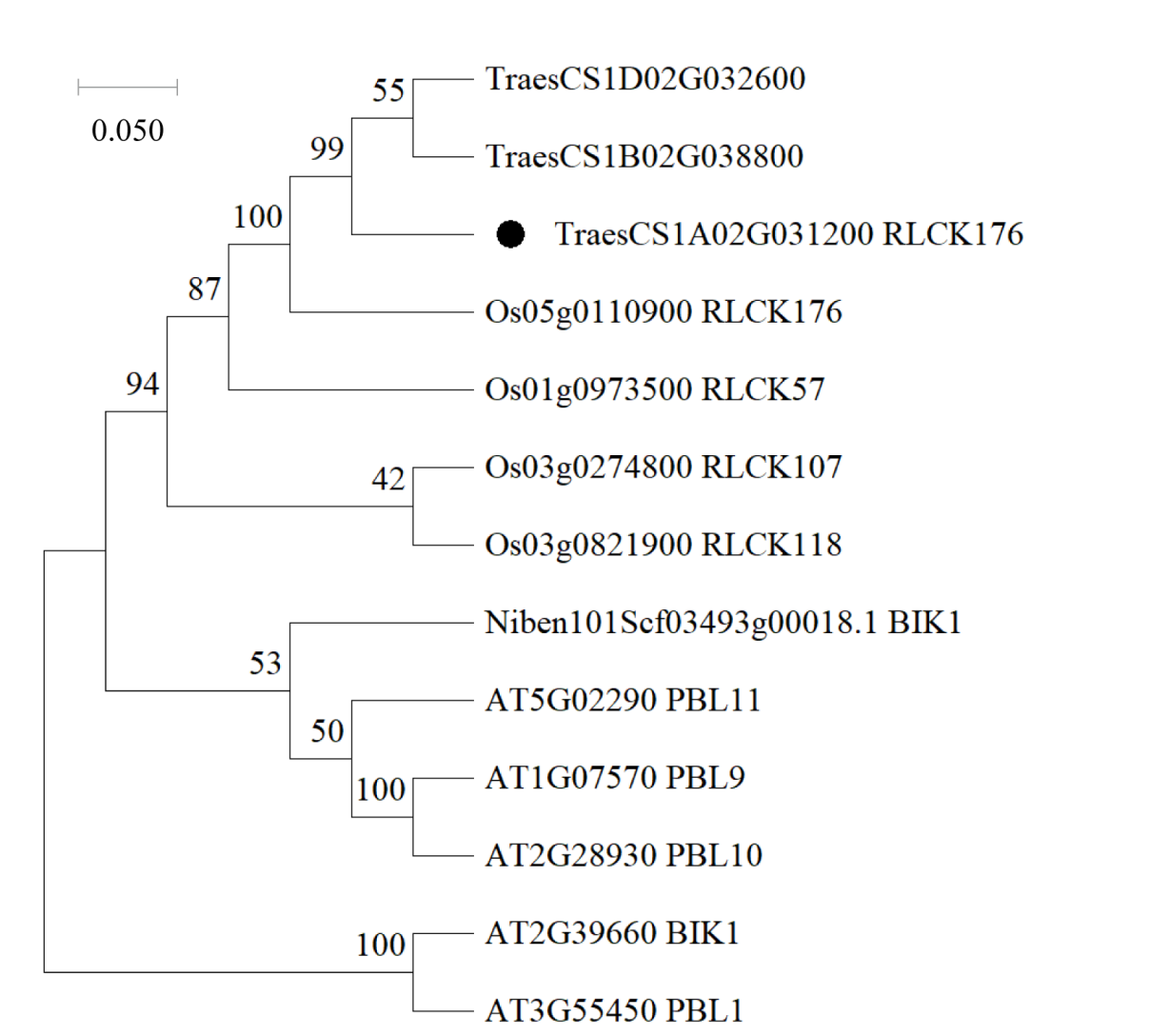


**Supplementary Figure 10. Phylogenetic analysis of RLCK homologs from wheat and other species.**

Ta, *Triticum* *aestivum*; Os, *Oryza* *sativa*; At, *Arabidopsis* *thaliana*; Nb, *Nicotiana* *benthamiana*. The MUSCLE algorithm within MEGA v6.0 was employed to perform multiple sequence alignments. A [phylogenetic tree](https://www.sciencedirect.com/topics/biochemistry-genetics-and-molecular-biology/phylogenetic-tree) was constructed using the pairwise deletion method, incorporating bootstrap values from 1,000 iterations.
