# Supplementary material for: Puccinia triticina Effector Pt3863 Targets and Subverts TaRLCK176 to Suppress Wheat Resistance to Leaf Rust
Source: Mol Plant Pathol. 2026 Jul 20;27(7):e70317. doi: 10.1111/mpp.70317 (PMC13382533; doi:10.1111/mpp.70317)
Supplement: Supplementary file 11 — Figure S11: Co‐immunoprecipitation validation of the interaction between Pt3863 and TaRLCK176. [file MPP-27-e70317-s014.docx]

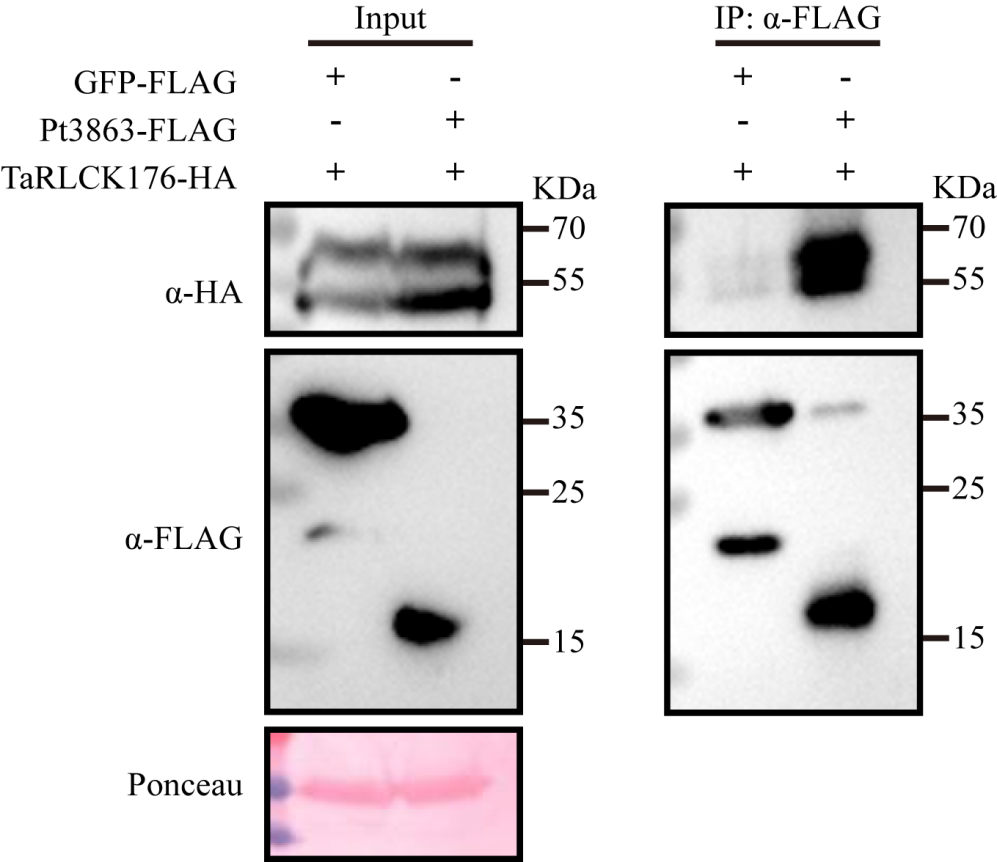


**Supplementary Figure 11.** **Co-IP validation of the interaction between Pt3863 and TaRLCK176.**

GFP-FLAG/TaRLCK176-HA and Pt3863-FLAG/TaRLCK176-HA were co-expressed in *N*. *benthamiana*. IP was performed using FLAG beads, and proteins were detected by western blot. Similar results were obtained from two independent experiments.
