# Supplementary material for: Puccinia triticina Effector Pt3863 Targets and Subverts TaRLCK176 to Suppress Wheat Resistance to Leaf Rust
Source: Mol Plant Pathol. 2026 Jul 20;27(7):e70317. doi: 10.1111/mpp.70317 (PMC13382533; doi:10.1111/mpp.70317)
Supplement: Supplementary file 12 — Figure S12: Split‐luciferase complementation validation of the interaction between Pt3863 and TaRLCK176. [file MPP-27-e70317-s018.docx]

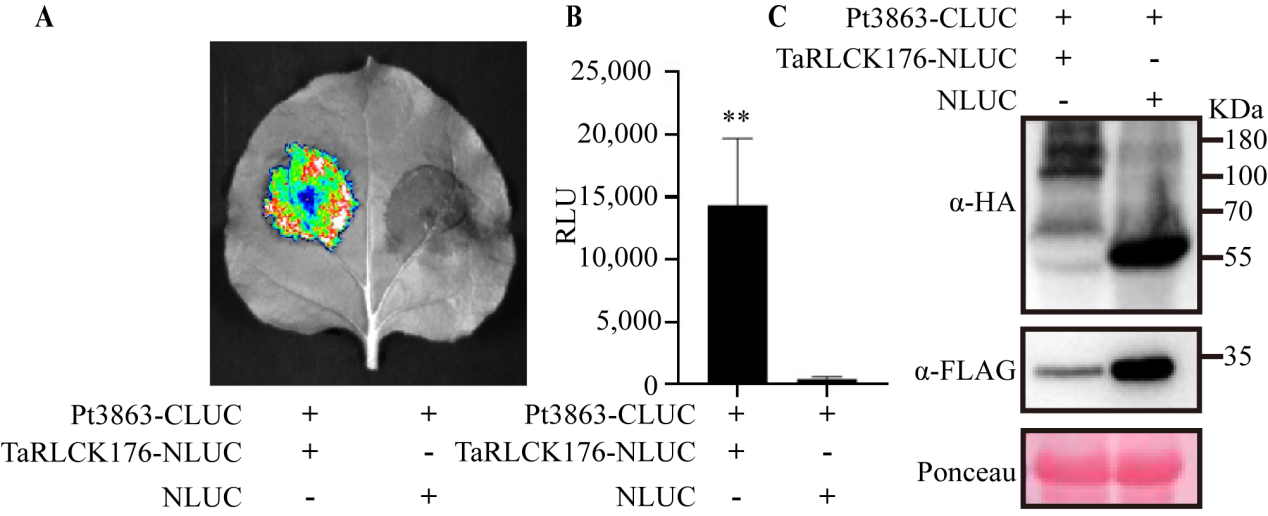


**Supplementary Figure 12. Split-Luc validation of the interaction between Pt3863 and TaRLCK176.**

A: Pt3863-Cluc and TaRLCK176-Nluc were co-expressed in *N.* *benthamiana* with Nluc as a control, and luminescence was visualized under a fluorometer 48 h later; B: The protein interaction intensity is shown by the relative luminescence unit (RLU) (Mean ± SD, n≥8, n represents sample number, ** *p* < 0.01, Student’s *t*-test); C: TaRLCK176-Nluc and Pt3863-Cluc were co-expressed in *N*. *benthamiana*, with Pt3863-Cluc and EV-Nluc as controls. The HA and FLAG tags were fused to the Nluc and Cluc vectors, respectively. Total proteins were extracted and detected by western blot.
