# Supplementary material for: Puccinia triticina Effector Pt3863 Targets and Subverts TaRLCK176 to Suppress Wheat Resistance to Leaf Rust
Source: Mol Plant Pathol. 2026 Jul 20;27(7):e70317. doi: 10.1111/mpp.70317 (PMC13382533; doi:10.1111/mpp.70317)
Supplement: Supplementary file 13 — Figure S13: Silencing of NbBIK1 inhibited flg22‐ and chitin‐induced reactive oxygen species (ROS), while transient expression of TaRLCK176 restored ROS accumulation. [file MPP-27-e70317-s016.docx]

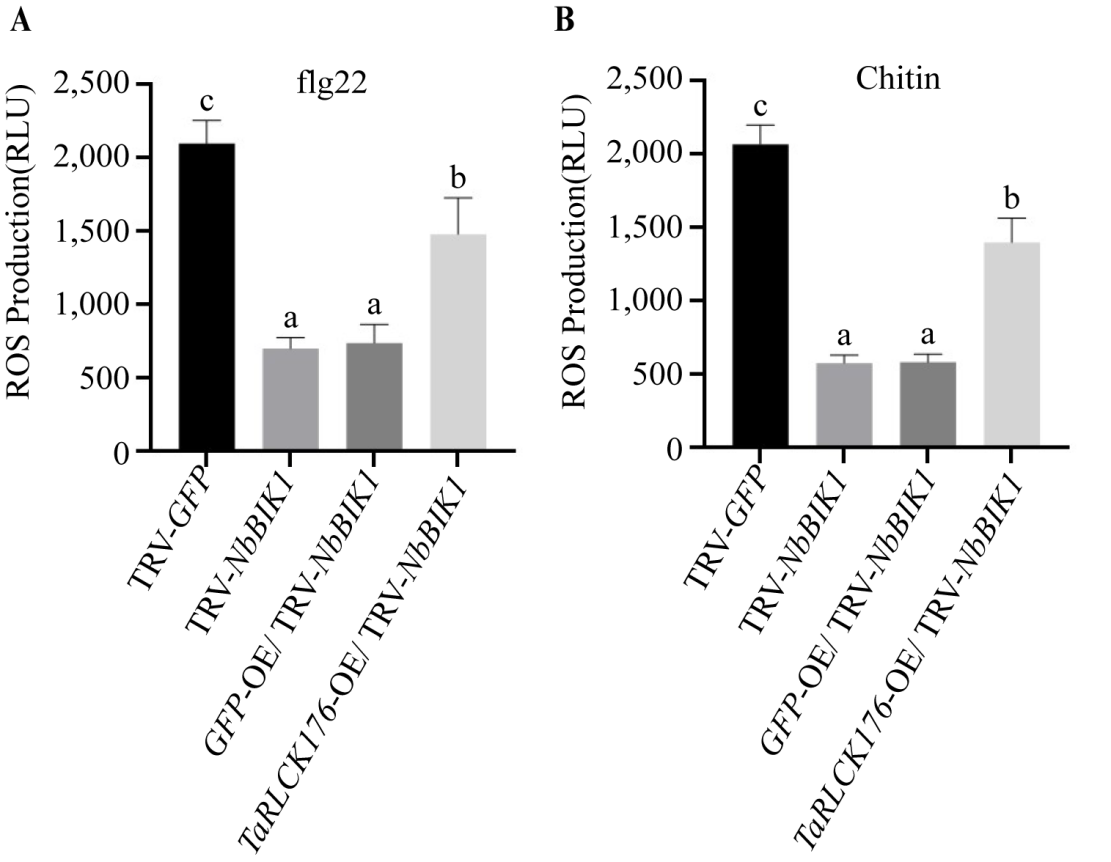


**Supplementary Figure 13. Silencing of *NbBIK1* inhibited flg22- and chitin-induced ROS, while transient expression of *TaRLCK176* restored ROS accumulation.**

Leaves of *N*. *benthamiana* with TRV-mediated gene silencing for 18 d and transient expression of *TaRLCK176* and *GFP* were treated with (A) 1 µM flg22 and (B) 10 µg/mL chitin for 10 min, respectively. Luminol-based chemiluminescence assays were performed using a microplate reader, and the experiments were repeated twice.
