# Supplementary material for: Puccinia triticina Effector Pt3863 Targets and Subverts TaRLCK176 to Suppress Wheat Resistance to Leaf Rust
Source: Mol Plant Pathol. 2026 Jul 20;27(7):e70317. doi: 10.1111/mpp.70317 (PMC13382533; doi:10.1111/mpp.70317)
Supplement: Supplementary file 14 — Figure S14: The silencing fragment and silencing efficiency of TaRLCK176. [file MPP-27-e70317-s020.docx]

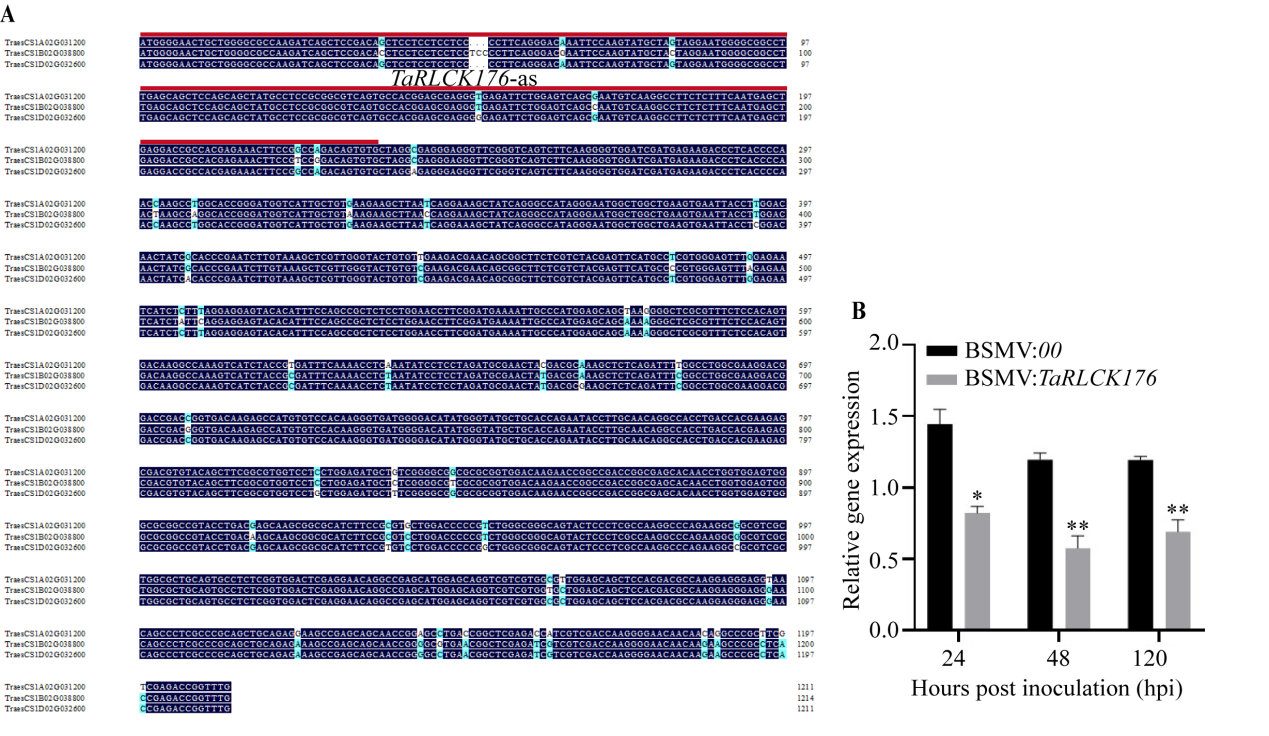


**Supplementary Figure 14. The silencing fragment and silencing efficiency of *TaRLCK176*.**

A: Red line highlighted region was used for BSMV-VIGS; B: The silencing efficiency of *TaRLCK176* was assessed using qRT-PCR in *TaRLCK176*-silenced plants
