# Supplementary material for: Puccinia triticina Effector Pt3863 Targets and Subverts TaRLCK176 to Suppress Wheat Resistance to Leaf Rust
Source: Mol Plant Pathol. 2026 Jul 20;27(7):e70317. doi: 10.1111/mpp.70317 (PMC13382533; doi:10.1111/mpp.70317)
Supplement: Supplementary file 15 — Figure S15: Expression analysis of TaRLCK176 genes in Pt3863 △SP ‐OE transgenic wheat during Puccinia triticina infection. [file MPP-27-e70317-s006.docx]

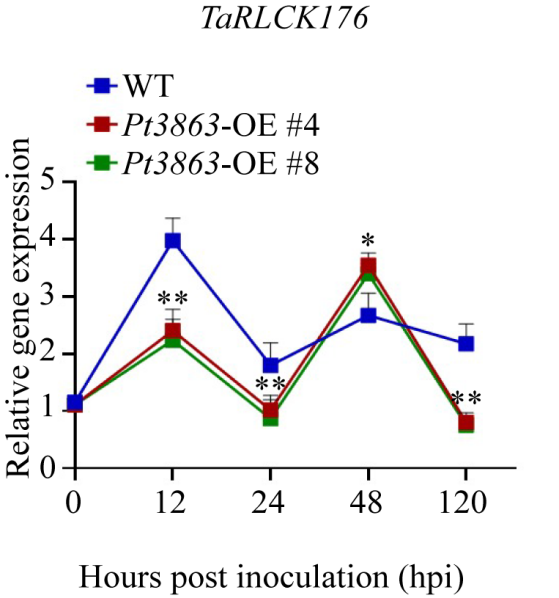


**Supplementary Figure 15. Expression analysis of *TaRLCK176* genes in *Pt3863^△sp^*-OE transgenic wheat during *Pt* infection.**

Statistical analysis was performed using Student’s *t*-test via Graphpad Prism v9.5, with three independent biological replicates per sample. All asterisks indicate significant differences compared with the WT group (* *p* < 0.05; ** *p* < 0.01).
