# Supplementary material for: Puccinia triticina Effector Pt3863 Targets and Subverts TaRLCK176 to Suppress Wheat Resistance to Leaf Rust
Source: Mol Plant Pathol. 2026 Jul 20;27(7):e70317. doi: 10.1111/mpp.70317 (PMC13382533; doi:10.1111/mpp.70317)
Supplement: Supplementary file 16 — Figure S16: TaRLCK176 exhibits no natural degradation. [file MPP-27-e70317-s012.docx]

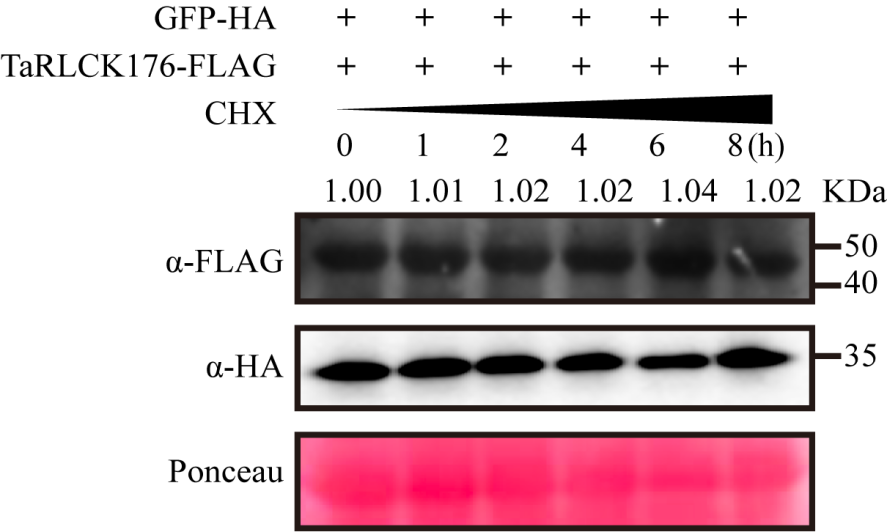


**Supplementary Figure 16.** **TaRLCK176 exhibits no natural degradation.**

GFP-HA/TaRLCK176-FLAG were co-injected into the expanded leaves of 4-week-old *N*. *benthamiana*. After 36 h, CHX was used for treatment at the indicated time points. Proteins were extracted for western blot detection. Similar results were obtained from two independent biological replicates.
