# Supplementary material for: Puccinia triticina Effector Pt3863 Targets and Subverts TaRLCK176 to Suppress Wheat Resistance to Leaf Rust
Source: Mol Plant Pathol. 2026 Jul 20;27(7):e70317. doi: 10.1111/mpp.70317 (PMC13382533; doi:10.1111/mpp.70317)
Supplement: Supplementary file 17 — Figure S17: Pt3863 affects the protein stability of TaRLCK176 through the ubiquitin mediated protein degradation pathway. [file MPP-27-e70317-s008.docx]

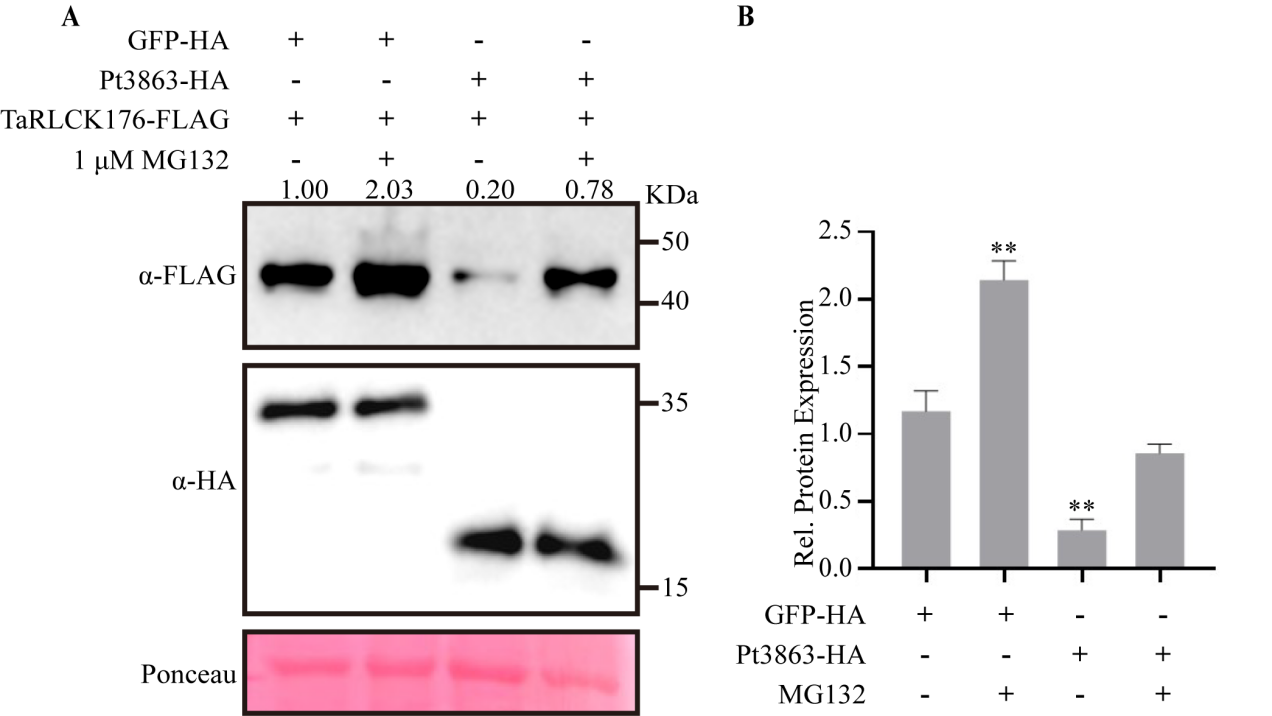


**Supplementary Figure 17. Pt3863 affects the protein stability of TaRLCK176 through the ubiquitin mediated protein degradation pathway.**

(A) and (B) Transient co-expression of TaRLCK176-FLAG with GFP-HA (control) or Pt3863-HA was performed in *N*. *benthamiana*. At 36 h post infiltration, all samples were treated with the 26S proteasome inhibitor MG132 for 6 h before protein extraction and Western blot detection. MG132 treatment increased the accumulation of TaRLCK176 in the control group and partially rescued the Pt3863-mediated degradation of TaRLCK176. These results indicate that Pt3863 destabilizes TaRLCK176 primarily through the ubiquitin-26S proteasome pathway. All experiments were repeated three times with similar results and data are mean ± SD of three independent experiments (** *p* < 0.01, Student’s *t*-test).
