# Supplementary material for: Puccinia triticina Effector Pt3863 Targets and Subverts TaRLCK176 to Suppress Wheat Resistance to Leaf Rust
Source: Mol Plant Pathol. 2026 Jul 20;27(7):e70317. doi: 10.1111/mpp.70317 (PMC13382533; doi:10.1111/mpp.70317)
Supplement: Supplementary file 18 — Figure S18: TaRLCK176 positively regulates chitin‐induced reactive oxygen species (ROS) accumulation. [file MPP-27-e70317-s021.docx]

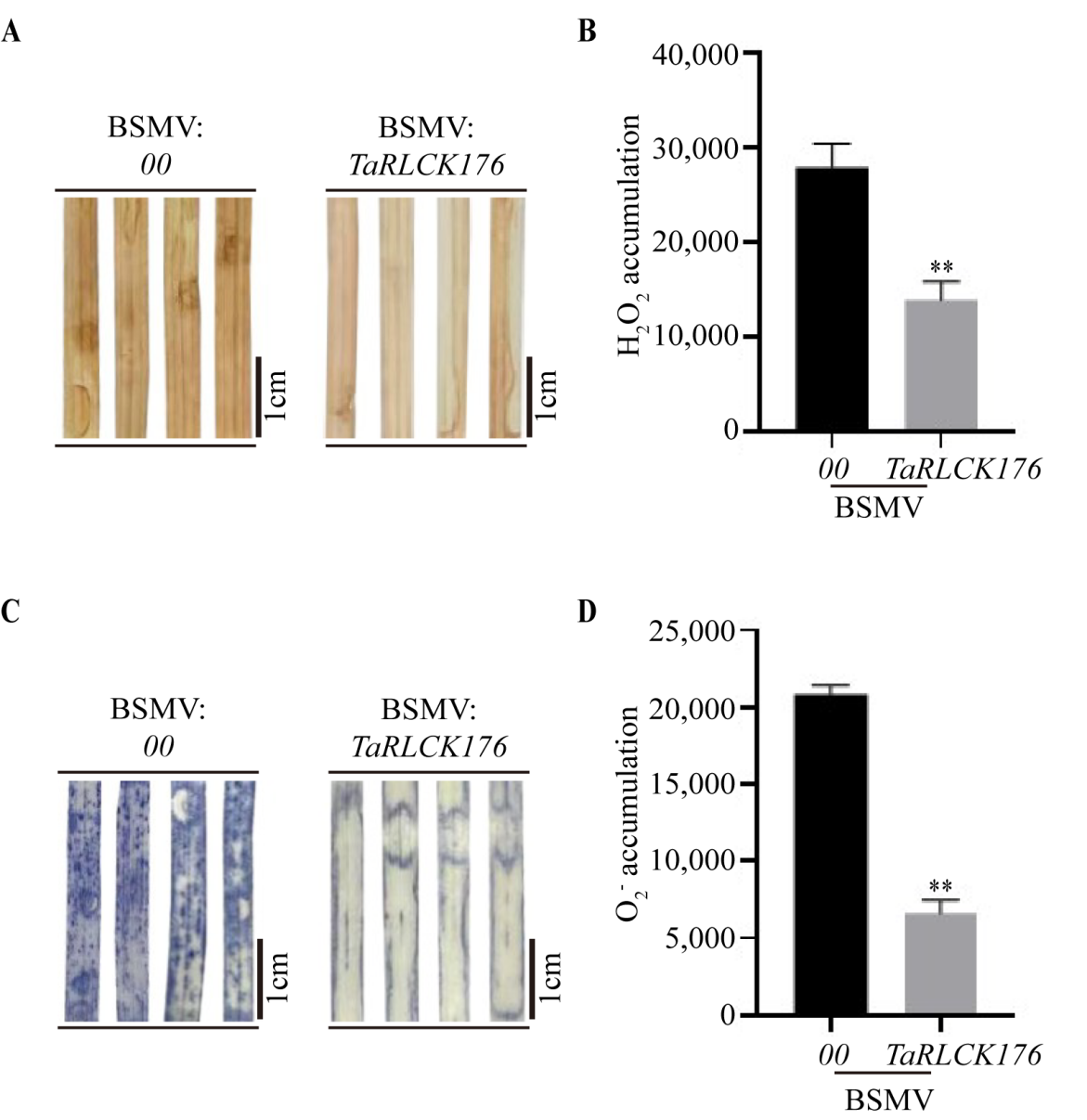


**Supplementary Figure 18. TaRLCK176 positively regulates chitin-induced ROS accumulation.**

(A) and (C) Samples from silenced lines were stained with DAB and NBT to detect hydrogen peroxide (H₂O₂) and superoxide anions (O₂⁻) at 2 h post inoculation (hpi) with chitin. (B) and (D) The percentages of H₂O₂ and O₂⁻ accumulation areas relative to the total injected leaf area are indicated by asterisks (** *p* < 0.01). Statistical analysis was performed using Student’s *t*-test in Prism v9.5 and three biological replicates were used for each sample
