# Supplementary material for: Puccinia triticina Effector Pt3863 Targets and Subverts TaRLCK176 to Suppress Wheat Resistance to Leaf Rust
Source: Mol Plant Pathol. 2026 Jul 20;27(7):e70317. doi: 10.1111/mpp.70317 (PMC13382533; doi:10.1111/mpp.70317)
Supplement: Supplementary file 19 — Figure S19: Sequence alignment of Pt3863 and Pst08755. [file MPP-27-e70317-s010.docx]

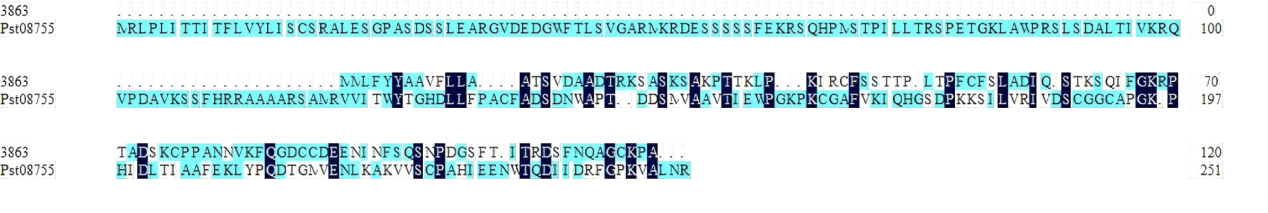


**Supplementary Figure 19. Sequence alignment of Pt3863 and Pst08755.**

The multiple sequence alignments within DNAMAN were employed to perform amino acid sequence alignment.
